# Unlocking Long‐Lived Charge‐Separated State in Pentamethine Cyanine Based on Anionic Hofmeister Effect for Photodynamic Immunotherapy

**DOI:** 10.1002/advs.77556

**Published:** 2026-09-11

**Authors:** Xuan Zhao, Lei Jiang, Yingyong Ni, Ting Wang, Junjun Wang, Shengyu Shi, Jianhua Yu, Zhou Lu, Xiaojiao Zhu, Hongping Zhou

**Affiliations:** ^1^ School of Chemistry and Chemical Engineering School of Materials Science and Engineering Key Laboratory of Structure and Functional Regulation of Hybrid Materials Ministry of Education Anhui University Hefei China; ^2^ School of Chemistry and Pharmaceutical Engineering Anhui Engineering Laboratory for Medicinal and Food Homologous Natural Resources Exploration Hefei Normal University Hefei China; ^3^ College of Biological and Food Engineering School of Chemical and Environmental Engineering Anhui Polytechnic University Wuhu China; ^4^ School of Physics and Electronic Information Anhui Normal University Wuhu China

**Keywords:** anionic Hofmeister effect, cyanine, electronic coupling, long‐lived charge‐separated state, photodynamic immunotherapy

## Abstract

The trade‐off between fluorescence imaging and photosensitization capability of pentamethine cyanine (Cy5) has greatly impeded its applications in phototherapy. Molecular design strategy has been the prevailing way to enhance its ROS generation efficiency, yet still unsatisfactory in the synergistic optimization of the synthesis procedure and the charge separation (CS) efficiency. Herein, by the incorporation of the stereo‐π anion **BPh_4_^−^** into a series of Cy5 skeletons, the ROS generation capability was enhanced by 8 times, and an efficient oxygen‐independent electron transfer pathway was evoked, unlocking the long‐lived charge‐separated state (CSs) and favoring photodynamic immunotherapy. The “End to End” stacked **Cy5‐BPh_4_** ensured weak electronic coupling (V) and a higher driving force for blocking charge recombination (CR), ultimately unfolding its efficient CS to facilitate subsequent electron transfer pathways. The superior ROS production in **Cy5‐BPh_4_** conferred potent phototoxicity against hypoxic cancer cells and elicited immunogenic cell death responses, achieving excellent antitumor efficacy in both primary and distant tumors. Overall, this work delivered a universal paradigm to endow cyanine‐PSs with long‐lived CSs for antitumor photodynamic immunotherapy.

## Graphical abstract

Based on the anionic Hofmeister effect, a universal strategy of incorporation of stereo‐*π* anion **BPh_4_^−^** into a series of Cy5 skeletons to unlock long‐lived charge‐separated states was reported, wherein the weak electronic coupling and low charge recombination driving force hinder the charge recombination process, elevating the oxygen‐independent ROS generation efficiency for efficient photodynamic immunotherapy.

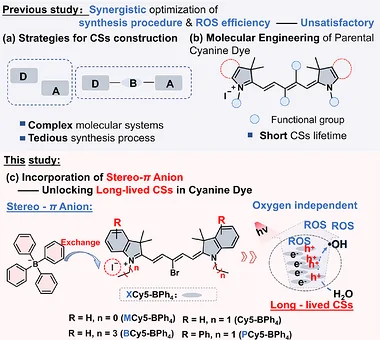

## 1 Introduction

**SCHEME 1:**
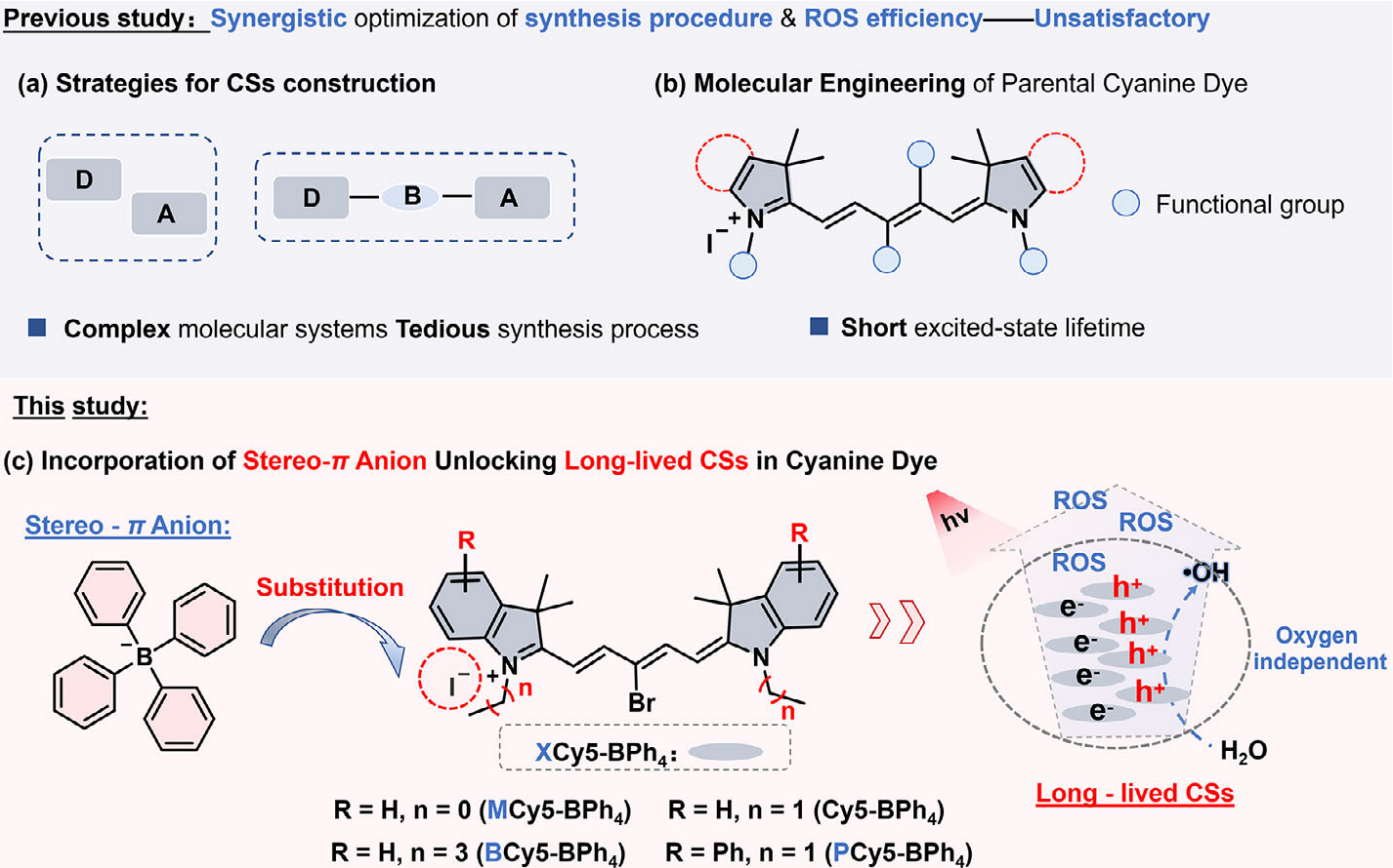
The schematic diagram of constructing long‐lived CSs (charge separated state)‐based PSs (photosensitizers) for obtaining type I ROS (reactive oxygen species). Previous works for (a) the strategies of constructing CSs through building D (donor)–A (acceptor) and D–B (bridge)–A systems. (b) molecular engineering based on cyanine dyes. (c) This work: Stereo‐*π* anion substitution strategy for unlocking long‐lived CSs in cyanine dyes.

The development of organic photosensitizers (PSs) with highly cytotoxic reactive oxygen species (ROS) (type I ROS, including O_2_^•−^, •OH, etc.) generation has attracted significant interest for their potential to eliminate hypoxic cancer cells and initiate a systemic immune response [1, 2, 3, 4, 5]. Most organic PSs exhibit suboptimal ROS generation efficiency due to the multiple decay processes of the excited states [6, 7, 8], which has stimulated tremendous efforts to control the photochemical process involved in ROS generation. Prevailing evidence has revealed that efficient charge separation (CS) can substantially elevate the chances for excited electrons and holes to participate in downstream photochemical processes [9, 10, 11, 12]. The strategies for fabricating the long‐lived charge‐separated state (CSs) mainly leverage interface energy level differences arising from the electron donor (D) and electron acceptor (A) in their binary blend system or a D‐ Bridge (B)—A system, allowing efficient charge transfer from D to A (Scheme 1a) [13, 14, 15, 16, 17, 18]. Obviously, these tactics inevitably encounter complex molecular systems as well as sophisticated synthetic routes, which are challenging and unfavorable for the development of highly efficient PSs for photodynamic immunotherapy. In this regard, there is an urgent need to develop alternative strategies for efficiently enhancing the ROS generation efficiency for ablating hypoxic tumors.

The anionic Hofmeister effect, reported by Franz Hofmeister in the late 1800s, initially applied to understand the anion‐modulated solubility and function of proteins, which have been extensively used to regulate molecular assembly behaviors over time via anion‐mediated structure modification [19, 20, 21]. The very new discovery by Zang's group has reported that specific anions are exploited for orchestrating phase transitions in isopolyoxometalate‐cation supramolecular assemblies [22]. As a matter of fact, the assembled mode of PSs driven by anions in the aqueous intracellular environment is highly correlated with their optical, physical, and chemical processes, which have been reported to promote fluorescence, photothermal, and photoacoustic performance, enhancing single oxygen production, and evoking the CS process [23, 24, 25, 26, 27]. Recently, Peng's group reported a longer lifetime (2.57 ns) of CSs in the assembled PSs by mitigating the charge recombination (CR) via the symmetry‐breaking CS [28]. In this regard, the anion‐mediated assembly regulation strategy based on the Hofmeister effect witnesses great advances in elevating the lifetime of CSs in the assembled PSs, a feat achieved while circumventing the tedious synthesis that constitutes a primary drawback of conventional approaches. Moreover, according to Marcus's theory, the lifetime of CSs is also closely correlated with electronic coupling (V) and the driving force referred to as ΔG_CR_, which were highly associated with molecular assembly behavior [29, 30, 31, 32]. However, the in‐depth investigation of the anion‐mediated strategy to facilitate the CS for robust ROS generation has never been reported.

Cyanine dyes, the classic commercial fluorophores, have attracted extensive research attention, but their short CSs lifetime hinders their photosensitization and ROS generation (Scheme 1b) [33]. Despite recognition of the anionic Hofmeister effect's role in optimizing cyanine assembly for the formation of CSs, its strategic use to modulate V and ΔG_CR_ for addressing the intrinsic limitation of short CS lifetimes has yet to be demonstrated. In this work, the anionic Hofmeister effect on the assembly of pentamethine cyanine (Cy5) was investigated to demonstrate how the anion‐mediated change of assembly behavior can be applied to regulate CSs. Importantly, by incorporation of the stereo‐π anion **BPh_4_^−^**, the long‐lived CSs were observed in a series of pentamethine cyanine (Cy5) skeletons to obtain efficient type‐I PSs for highly efficient photodynamic immunotherapy (Scheme 1c). Especially, the **Cy5‐BPh_4_** presented the “End to End” stacking due to the stereo‐*π* anion **BPh_4_^−^**, accomplishing a long‐lived CSs (9.39 ns) for boosting photo‐induced O_2_^•−^ and •OH generation compared to that of the Cy5 incorporated with the planar anions. Transient absorbance spectra of **Cy5‐BPh_4_** revealed that the cascade electron transfer process from localized CSs to delocalized CSs. Furthermore, owing to the relatively weak V, **Cy5‐BPh_4_** exhibited the lower ΔG_CR_ for prolonging the lifetime of delocalized CSs, guaranteeing the efficient electron transfer to produce superior O_2_^•−^ and •OH. Benefited from that, **Cy5‐BPh_4_** not only enabled server mitochondria oxidative stress‐induced cell apoptosis, but also induced immunogenic cell death (ICD), during which the derived systemic immune response effectively eradicated primary and distant tumors.

## 2 Materials and Methods

### 2.1 Chemicals and Materials

The chemical reagents including 2,3,3‐trimethyl‐3H‐indole, iodine ethane, mucobromic acid, aniline, 1,3‐bisdicyanovinylindan, silver 4‐methylbenzenesulfonate, sodium tetraphenylboron, sodium hydroxide, sodium acetate, anhydrous sodium sulfate, toluene, methanol, ethanol, acetic anhydride, ether, and dichloromethane were purchased from Aladdin Co., Ltd, Macklin Co., Ltd, and so forth. The other solvents as well as reagents were obtained from the company (Absin B.D. Thermo, BestBio, Sigma‐Aldrich, MKBio, Co., Ltd), such as 2′,7′‐dichlorofluorescin diacetate (DCFH‐DA), hydroxyphenyl fluorescein (HPF), dihydrorhodamine 123 (DHR123), 5,5‐dimethyl‐1‐pyrroline N‐oxide (DMPO), Mito tracker Green, ROS‐ID Hypoxia/Oxidative Stress Detection Kit, dihydroethidium (DHE), Mitochondrial Membrane Potential Assay Kit (JC‐10), Calcein‐AM and propidium iodide (PI), DAPI, Annexin V‐FITC/PI detection kit, IFN‐*γ* ELISA kit, IL‐12 ELISA kit, and TNF‐*α* ELISA kit, and so on.

### 2.2 Instrumentation

The characteristics of ^1^H and ^13^C NMR spectra for **Cy5‐I**, **Cy5‐OTs**, **Cy5‐FCN**, **Cy5‐BPh_4,_ MCy5‐BPh_4_**, **BCy5‐BPh_4_**, and **PCy5‐BPh_4_** were obtained from a JNM‐ECZ400S spectrometer using DMSO‐*d*_6_ as solvent and tetramethyl silane (TMS) as reference at room temperature. Electrospray ionization mass spectrometric (ESI‐MS) data were recorded on Thermo Fisher Scientific LTQ‐Orbitrap XL mass spectrometer. The UV–vis spectra were captured on UV‐3900 spectrophotometer and the fluorescence spectra were detected by Hitachi F‐4500 fluorescence spectrophotometer. Dynamic light scattering (DLS) assay was measured with Nano‐zs90. Transmission electron microscopy (TEM) as well as high‐resolution TEM (HR‐TEM) images were performed by a JEM‐2100F and JEM‐F200 field‐emission transmission electron microscope. Electron paramagnetic resonance (EPR) spectra were recorded utilizing the Bruker Nano x‐band spectrometer (Magnettech EPR500). The electrochemical measurements were conducted on an electrochemical workstation (Chenhua CHI‐660E) in a standard three‐electrode cell. Confocal laser scanning microscope (CLSM) (Laica TCS SP8 DIVE/SP8 DIVE) with 10/40/63 ×  objective lens was used to obtain cell images, and ImageJ software was leveraged to process the pictures.

### 2.3 Synthesis of Cy5‐FCN

The 0.094 g (482.47 g/mol, 0.20 mmol) of 1,3‐bisdicyanovinylindan was dissolved in an aqueous solution, and then the pH value of the solution was adjusted to around 9–10 utilizing NaOH solution (0.01 mol/L). Subsequently, a solution of **Cy5‐I** (0.1 g, 617.41 g/mol, 0.16 mmol) in 20 mL of methanol was added to the above mixture and further stirred at 65°C for 24 h. Upon reaction completion, methanol was evaporated under reduced pressure, filtered to obtain the crude product, and washed with aqueous solution, then recrystallized from 20 mL of ethanol to afford **Cy5‐FCN** as a dark solid in 73.8% yield.

### 2.4 Synthesis of Cy5‐BPh_4_

The 0.1 g (617.41 g/mol, 0.16 mmol) of **Cy5‐I** and 0.058 g (342.22 g/mol, 0.17 mmol) of sodium tetraphenylboron were dissolved in 20.0 mL methanol as well as refluxed at 65°C for 4 h. After the reaction finished, the mixture was filtered and recrystallized from 15 mL ethanol to afford **Cy5‐BPh_4_** as a dark blue solid in 79.4% yield.

### 2.5 Preparation of Solution

The seven targeted compounds **Cy5‐X** (donated as **Cy5‐I**, **Cy5‐OTs**, **Cy5‐FCN**, **Cy5‐BPh_4_**, **MCy5‐BPh_4_**, **BCy5‐BPh_4_**, and **PCy5‐BPh_4_**) were initially dissolved in DMSO to obtain stock solutions at a concentration of 10^−3^ M, diluting with redistilled water/ DMSO to 10^−5^ M for the subsequent testing.

### 2.6 Total ROS Detection

DCFH‐DA was leveraged for detecting the production of total ROS. Details, **Cy5‐I**, **Cy5‐FCN**, or **Cy5‐BPh_4_** (10 µM) in H_2_O was mixed with DCFH‐DA (100 µM), followed by exposure to light for 0–30 s (660 nm laser, 100 mW cm^−2^), respectively. The DCF fluorescence intensity at 525 nm in each group was measured using a fluorescence spectrophotometer.

### 2.7 ^1^O_2_ Detection

ABDA was used to quantify the ^1^O_2_ generation of **Cy5‐X**. The concentrations of **Cy5‐X** and ABDA were retained at 10 and 100 µM, respectively. The absorption spectra of ABDA in each group were promptly recorded under light conditions for 0–100 s.

### 2.8 O_2_^•−^ and •OH Detection

DHR123 and HPF were utilized as indicators for evaluating O_2_^•−^ and •OH generation of **Cy5‐X** upon irradiation, respectively. Taking DHR123 for example, its solution was mixed with **Cy5‐X** in redistilled water (2 mL) and then illuminated with a 660 nm laser (100 mW cm^−2^) for 30 s (time interval: 3 s). Subsequently, the fluorescence spectrometer to monitor the fluorescence intensity of DHR123.

### 2.9 O_2_^•−^ and •OH Detection via EPR Assay

EPR measurement was used to detect the generation of O_2_^•−^ and •OH, employing DMPO as a spin‐trap agent. Briefly, a DMSO/H_2_O solution containing 0.1 mM **Cy5‐X** was mixed with 5 mM DMPO in air or N_2_ atmosphere, respectively, and then illuminated for 1 min using 660 nm laser (100 mW cm^−2^).

### 2.10 Mass Spectra Measurement

The DMPO was added in **Cy5‐FCN** or **Cy5‐BPh_4_** solution in H_2_^18^O (0.5 mL), respectively. Then the sample solutions were illuminated with a 660 nm laser (100 mW cm^−2^) for 15 min, and the above samples were used for mass spectra analysis.

### 2.11 Transient Absorption Spectroscopy

All transient absorption spectrum (TAS) were acquired using a transmission pump‐probe (UV/vis pump‐broadband supercontinuum probe) setup, equipped with a regenerative amplified Ti: sapphire laser system (SpitFire Ace, Spectra Physics; center wavelength: 800 nm, pulse energy: 6 mJ, pulse width: ∼80 fs). Briefly, the regenerative amplifier output was split: one beam generated wavelength‐tunable pump light for TAS via a TOPAS and was chopped at 500 Hz, while the other passed through an optical delay line (0–8 ns) and was focused into a CaF_2_ crystal for producing white light continuum (WLC) as the probe. Solution samples were excited at 650 nm as well as probed with a WLC pulse spanning 350–700 nm.

### 2.12 Theoretical Calculation

The Gaussian 16 suite of programs was leveraged to optimize the configurations. Structural optimization and frequency were conducted using the M06‐2X functional with the 6–311 G (d, p) basis set (GD 3). Time‐dependent DFT was also carried out at the same level [34].

### 2.13 Cell and Culture Conditions

The 4T1 mouse breast and Hep G2 human hepatoma cancer cells were cultured in DMEM medium supplemented with 10% FBS and antibiotics (100 U/mL penicillin, 100 µg/mL streptomycin) at 37°C under 5% CO_2_. For imaging purposes, the cells were seeded onto 15 mm × 15 mm confocal dishes and incubated for 12–24 h.

### 2.14 Cytotoxicity Assays

The 4T1 and Hep G2 cells were plated in 96‐well plates at 1 × 10^4^ cells per well and incubated at 37°C. For investigating the cytotoxicity, **Cy5‐BPh_4_** with varying concentrations was added to each well under normoxic or hypoxic conditions, then incubated for an additional 30 min with or without light irradiation. Following this, the cell culture media were replaced with 200 µL of fresh medium, accompanied by 5 mg/mL MTT solution (10 µL). After 4 h of incubation, the medium was removed carefully, prior to adding 100 µL DMSO into each well. Gently slackening the plate to dissolve the formazan, and finally, the absorbance was read at 570 nm on a microplate reader.

### 2.15 Intracellular Hypoxic Construction

The cancer cells were pre‐cultured in N_2_ atmosphere for 1 h prior to incubation with **Cy5‐BPh_4_** for mimicking the hypoxic environment. Subsequently, the ROS‐ID Hypoxia/Oxidative Stress Detection Kit served as a specific probe to assess hypoxic conditions in 4T1 and Hep G2 cells, with mean fluorescence intensities quantified using ImageJ software.

### 2.16 Intracellular Radical Detection

DHE and HPF were utilized for detecting intracellular O_2_^•−^ and •OH, respectively. The cells were incubated with 5 µM **Cy5‐BPh_4_** for 10 min under normoxic or hypoxic conditions to allow uptake, respectively, followed by incubation with 10 µM DHE and 2 µM HPF for another 15 min. Thereafter, the cells were washed with PBS three times and irradiated with light for 5 min. The fluorescence of DHE and HPF was observed using CLSM, with excitation at 535 and 488 nm, which were monitored at 600–650 and 500–520 nm, respectively.

### 2.17 Live/Dead Cell Stain Assay

After 4T1 cells were preincubated with **Cy5‐BPh_4_** (5 µM) for 10 min under normoxic or hypoxic conditions, stained with 5 µL Calcein‐AM and PI for 15 min, respectively. CLSM was used to visualize cell death, with excitation wavelengths of 488 nm and 535 nm, respectively. The green channel of Calcein‐AM was detected with an emission wavelength range of 505–530 nm (donated as living cells), and the red channel of PI was detected with an emission wavelength range of 600–700 nm (donated as dead cells).

### 2.18 Damage‐Associated Molecular Patterns (DAMPs) From 4T1 Cells

CRT expression, release of HMGB1 and ATP were analyzed to evaluate the DAMPs from cell lines. 4T1 cells were incubated with **Cy5‐BPh_4_** (5 µM) at 37°C, followed by exposure to 10 min of 660 nm laser irradiation.

#### 2.18.1 Detection of the Release of Intracellular CRT and HMGB1

After 6 h of incubation, cells were washed three times with PBS, fixed in 4% paraformaldehyde on ice for 20 min, and then incubated overnight at 4°C with anti‐CRT and anti‐HMGB1 antibodies. Subsequently, Alexa Fluor 488‐conjugated secondary antibody was leveraged to mark cells at room temperature for 2 h. CRT expression as well as HMGB1 release were visualized via CLSM.

#### 2.18.2 Detection of the Secretion of Extracellular HMGB1 and ATP

Following a 6 h incubation, cell culture supernatants were harvested for the quantitative analysis of extracellular HMGB1 and ATP via commercial ELISA and bioluminescence assay kits, respectively.

### 2.19 In Vivo Photodynamic Efficacy

The 6‐week‐old female Balb/c mice (Mouse certificate number: 20260225Abzz010500000009) were obtained from Hangzhou Ziyuan Experimental Animal Technology Co., LTD, which was approved by the Institutional Animal Care and Use Committee of Anhui University (No: IACUC(AHU)‐2024‐054). The 4T1‐bearing mice with appropriate tumor size were selected and split into four groups as (I) Control (PBS), (II) Control + L (PBS + Light), (III) **Cy5‐BPh_4_**, and (IV) **Cy5‐BPh_4_** + **L** (**Cy5‐BPh_4_ + Light**) for evaluating the photodynamic and immune therapy efficacy, in which **Cy5‐BPh_4_** solutions (100 µM, 50 µL) were intratumorally injected on days 0 and 3. Each group comprised three mice. After 4 h post‐injection, the tumor regions were exposed to 660 nm laser irradiation at a power density of 100 mW cm^−2^ for 20 min. The mice's body weight and tumor volume were monitored each 3 days throughout the whole experimental period. After 15 days, the mice in each group were killed, and the tumors in the primary and distant sites were separated and photographed. Therein, the tumor volume was determined by volume = 1/2×length×width [2]. The fresh tumor tissues and other normal organs (heart, liver, spleen, lung, and kidney) were fixed in 4% paraformaldehyde and stained with H&E, followed by examination under a light microscope for antitumor and toxicity analysis.

### 2.20 Analysis of Immune Indicators

To assess the effectiveness of ICD in vivo, the tumors in the primary and distant, spleen, and lymph nodes of the mice in each group were collected after 15 days of treatment and prepared as single‐cell suspensions. The single‐cell suspensions were stained with anti‐CD45, anti‐CD3 and anti‐CD8 or anti‐CD11c, anti‐CD80, as well as anti‐CD86, washed with 1 × PBS, and analyzed by flow cytometry. Transwell medium was collected for ELISA quantification of TNF‐α, IFN‐γ, and IL‐12.

### 2.21 Statistical Analysis

GraphPad Prism 10.6 and SPSS were employed for data analysis. Data are expressed as mean ± SD, with experimental replicates (n) detailed in the figure legends. Comparisons between two groups were performed using two‐tailed unpaired Student's *t*‐test, whereas one‐way ANOVA (SPSS) was used for multiple comparisons. The statistical significance was donated as ^*^ *p* < 0.05, ^**^ *p* < 0.01, ^***^ *p* < 0.001.

## 3 Results and Discussion

### 3.1 Molecular Design and Synthesis

As a traditional parent dye, Cy5 possesses near‐infrared (NIR) absorbance and emission, as well as a high extinction coefficient, enabling effective photon capture in deep tissues [35, 36, 37]. Also, assembled cyanine derivatives hold a lower reorganization energy, which was beneficial for forming CSs [38, 39]. Therefore, Cy5 was selected as the cation scaffold, as well as, distinct *ππ‐*anion of the stereo type (**BPh_4_** ^−^) and planar type (**OTs^−^** and **FCN^−^**) were introduced to replace the heavy atom (I^−^), yielding three different PSs named **Cy5‐OTs**, **Cy5‐FCN**, and **Cy5‐BPh_4_**, respectively. The synthetic routes of the three targeted compounds and intermediates were displayed in Scheme S1 with the corresponding characterization using ^1^H NMR, ^13^C NMR, and ESI‐MS (Figures S1–S16). The single crystals of **Cy5‐FCN** (CCDC: 2478812) and **Cy5‐BPh_4_** (CCDC: 2478811) were measured by X‐ray diffraction and analyzed crystallographically with the specific crystal data in Table S1.

### 3.2 Molecular Self‐Assembly and Radical Generation

**FIGURE 1:**
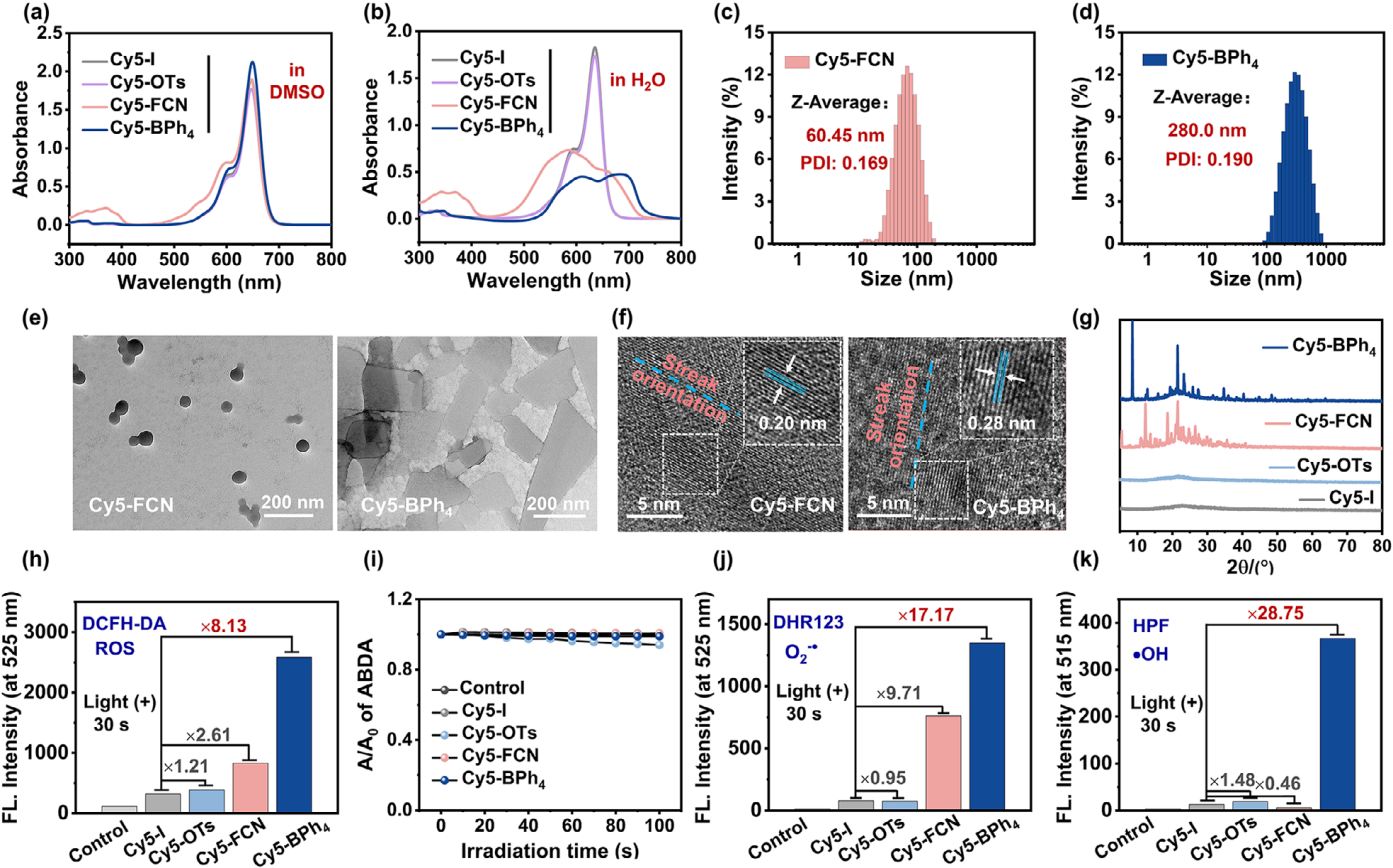
Molecular self‐assembly and photo‐induced radical generation. The absorption spectra of **Cy5‐X** (10 µM) (X = I, OTs, FCN, and BPh_4_) in (a) DMSO and (b) redistilled water. The dynamic light scattering (DLS) analysis of (c) **Cy5‐FCN** (10 µM) and (d) **Cy5‐BPh_4_** (10 µM) in redistilled water. (e) The representative transmission electron microscope (TEM) images of **Cy5‐FCN** (10 µM) and **Cy5‐BPh_4_** (10 µM). Scale bar: 200 nm. (f) The high‐resolution TEM images of the self‐assembled structures for **Cy5‐FCN** (10 µM) and **Cy5‐BPh_4_** (10 µM), with TEM lattice fringes and lattice distance. Scale bar: 5 nm. (g) Powder X‐ray diffraction (XRD) patterns of **Cy5‐X**. Comparison of fluorescence enhancement of (h) DCFH‐DA (100 µM), (j) DHR123 (2 µM), and (k) HPF (2 µM) in the presence of **Cy5‐X** (10 µM) following 30 s laser irradiation (660 nm, 100 mW cm^−2^). Solvent: redistilled water. (i) Decomposition rates of ABDA (100 µM) in the presence of **Cy5‐X** (10 µM) at different times under laser illumination (660 nm, 100 mW cm^−2^). Solvent: redistilled water.

For exploring the self‐assembly behavior of the obtained PSs, a series of experiments were performed. Initially, DMSO and redistilled water were selected as good and poor solvents to investigate the optical characteristics of PSs, respectively. As shown in Figure 1a, both compounds displayed a comparable absorption peak at around 650 nm with a shoulder at ∼590 nm in DMSO, which was consistent with the characteristic absorption of the prototypical Cy5 molecular skeleton. Nevertheless, significantly broadened and decreased absorption were captured when **Cy5‐FCN** and **Cy5‐BPh_4_** were in redistilled water. It was noted that the difference in the maximum absorption was observed as a red shift for **Cy5‐BPh_4_** and a blue shift for **Cy5‐FCN**, which might be implicated in forming aggregates with distinct self‐assembly behaviors (Figure 1b). Fluorescence emission spectra separately revealed the weaker fluorescence of **Cy5‐FCN** and **Cy5‐BPh_4_** in aqueous solution with lower fluorescence quantum yields (*Φ_f_*) of 0.53% and 1.57% in contrast to those in DMSO (**Cy5‐FCN**: 12.21%; **Cy5‐BPh_4_**: 12.11%), which was primarily attributed to the aggregation‐induced fluorescence quenching (Figure S29).

Subsequently, the dynamic light scattering (DLS) assay and transmission electron microscope (TEM) were implemented to explore the specific self‐assembly behaviors of the obtained PSs driven by Hofmeister effect regulation. In comparison with **Cy5‐I** and **Cy5‐OTs**, the discernible particle size distribution and formed nanostructures of **Cy5‐FCN** (∼60 nm) and **Cy5‐BPh_4_** (∼280 nm) in redistilled water, unveiling the efficient formation of molecular aggregates (Figure 1c,d; Figure S30). Notably, **Cy5‐FCN** and **Cy5‐BPh_4_** presented spherical aggregates as well as flake‐like aggregates, respectively, preliminarily explaining the differences in their absorbance behavior in redistilled water and revealing the anionic Hofmeister effect‐mediated assembly change (Figure 1e). In addition, the high‐resolution TEM images and powder X‐ray diffraction (XRD) pattern showcased the distinct crystallinity, in which the prominent crystallization peaks were characterized, as well as clear *d*‐spacing values of **Cy5‐FCN** and **Cy5‐BPh_4_** were 0.20 nm and 0.28 nm, respectively (Figure 1f,g). The findings above demonstrated that the modulating counter‐anions to **Cy5** could induce efficient molecular self‐assembly in an aqueous environment.

Given the quenched fluorescence on assembled **Cy5‐FCN** and **Cy5‐BPh_4_**, the nonradiative deactivation pathways might occur to produce ROS. At first, the probe DCFH‐DA was adopted as an indicator to detect the total ROS, as it exhibited bright green fluorescence of DCF at around 525 nm after being oxidized by ROS. As shown in Figure 1h and Figure S31, in sharp contrast to nonaggregated **Cy5‐I** and **Cy5‐OTs**, obvious fluorescence enhancement at about 525 nm was observed in assembled **Cy5‐FCN** and **Cy5‐BPh_4_** groups within 30 s irradiation (660 nm, 100 mW cm^−2^) in aqueous solution, manifesting the great ROS generation ability of the self‐assembled PSs. Of note, the remarkable fluorescence enhancement of DCF (8.13‐fold compared with **Cy5‐I**) was recorded in the **Cy5‐BPh_4_** group, while only a 2.61‐fold increase was observed in the **Cy5‐FCN** group, proclaiming the superior photoinduced ROS‐generating ability of **Cy5‐BPh_4_**.

Further, the specific ROS produced by assembled **Cy5‐FCN** and **Cy5‐BPh_4_** under 660 nm laser irradiation were evaluated by utilizing ABDA (for ^1^O_2_), DHR123 (for O_2_^•−^), and HPF (•OH) as indicators, respectively. Compared with the control, all the groups showed a tiny impaired absorption of ABDA, indicating negligible ^1^O_2_ generation in the obtained PSs within 100 s irradiation (Figure 1i; Figure S32). Conversely, as depicted in Figure 1j and Figure S33, upon 660 nm laser illumination for 30 s, the significantly increased fluorescence of DHR 123 was captured both in **Cy5‐FCN** and **Cy5‐BPh_4_**, demonstrating the prominent O_2_^•−^ generation ability in the aggregated PSs. Typically, the fluorescence intensity of DHR123 and HPF in the **Cy5‐BPh_4_** group displayed a higher degree of enhancement (Figure 1k; Figure S34) than that of assembled **Cy5‐FCN** with a planar anion, suggesting that the incorporation of stereo‐*π* anion of **BPh_4_^−^** was more beneficial to unlock the photo‐induced O_2_^•−^ and •OH. These stark differences in radical generation emphasized the anionic Hofmeister effect as a potent strategy for programmable design of efficient type I cyanine‐based PSs.

### 3.3 Universality of Stereo‐*π* Anion Driven Photo‐Induced Radical Yield

**FIGURE 2:**
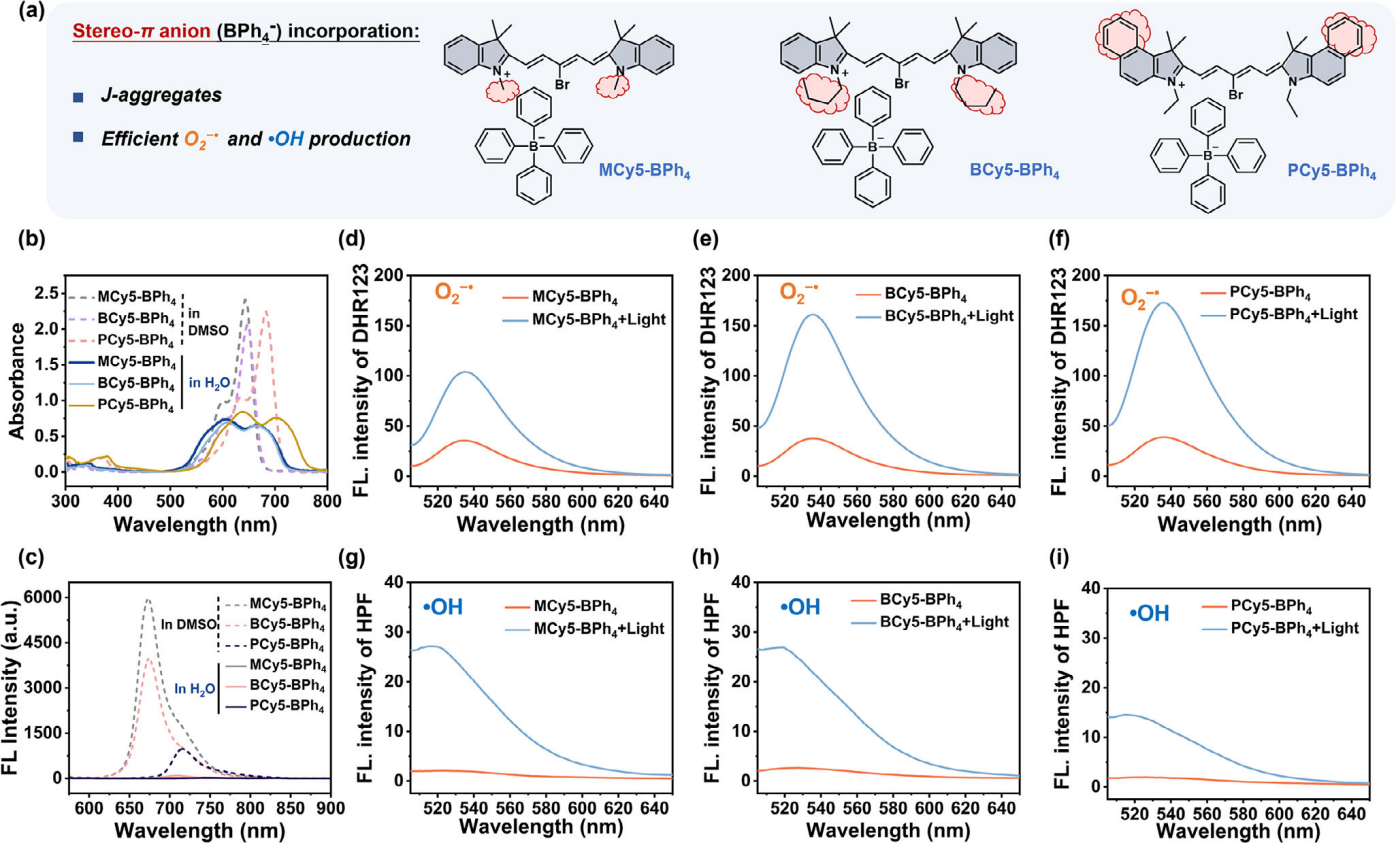
Universality of stereo‐*π* anion‐driven molecule assembly and radical generation. (a) Chemical structures of alternative cyanine dyes after integrating **BPh_4_** ^−^. (b) The absorption and (c) fluorescence spectra of **MCy5‐BPh_4_** (10 µM), **BCy5‐BPh_4_** (10 µM), and **PCy5‐BPh_4_** (10 µM) in DMSO and redistilled water, respectively. The fluorescence spectra of DHR123 (2 µM) after incubating with (d) **MCy5‐BPh_4_** (10 µM), (e) **BCy5‐BPh_4_** (10 µM), and (f) **PCy5‐BPh_4_** (10 µM) under 660 nm laser irradiation (100 mW cm^−2^) within 60 s for detecting O_2_^•−^ generation, respectively. The fluorescence spectra of HPF (2 µM) after incubating with (g) **MCy5‐BPh_4_** (10 µM), (h) **BCy5‐BPh_4_** (10 µM), and (i) **PCy5‐BPh_4_** (10 µM) under 660 nm laser irradiation (100 mW cm^−2^) within 60 s for detecting •OH generation, respectively.

To this end, the stereo‐*π* anion strategy was applied to other cyanine dyes such as **MCy5‐BPh_4_**, **BCy5‐BPh_4_**, and **PCy5‐BPh_4_**, respectively (Figure 2a). First, all PSs were characterized by ^1^H NMR, ^13^C NMR, and ESI‐MS (Figures S17–S28), and their optical properties were tested immediately. Meaningfully, **MCy5‐BPh_4_**, **BCy5‐BPh_4_**, and **PCy5‐BPh_4_** displayed similar optical properties to **Cy5‐BPh_4_** in redistilled water, demonstrated the alike aggregates behavior (Figure 2b,c). Moreover, as displayed in Figure 2d–i and Figure S35, upon light irradiation, **MCy5‐BPh_4_**, **BCy5‐BPh_4_**, and **PCy5‐BPh_4_** also exhibited the unlocking of photo‐induced O_2_^•−^ and •OH generation compared with the cyanine PSs without **BPh_4_** ^−^ modification, underscoring the universality of stereo‐*π* anion **BPh_4_^−^** in cyanine for constructing efficient type I PSs.

### 3.4 Water Oxidation Process and Electron Transfer

**FIGURE 3:**
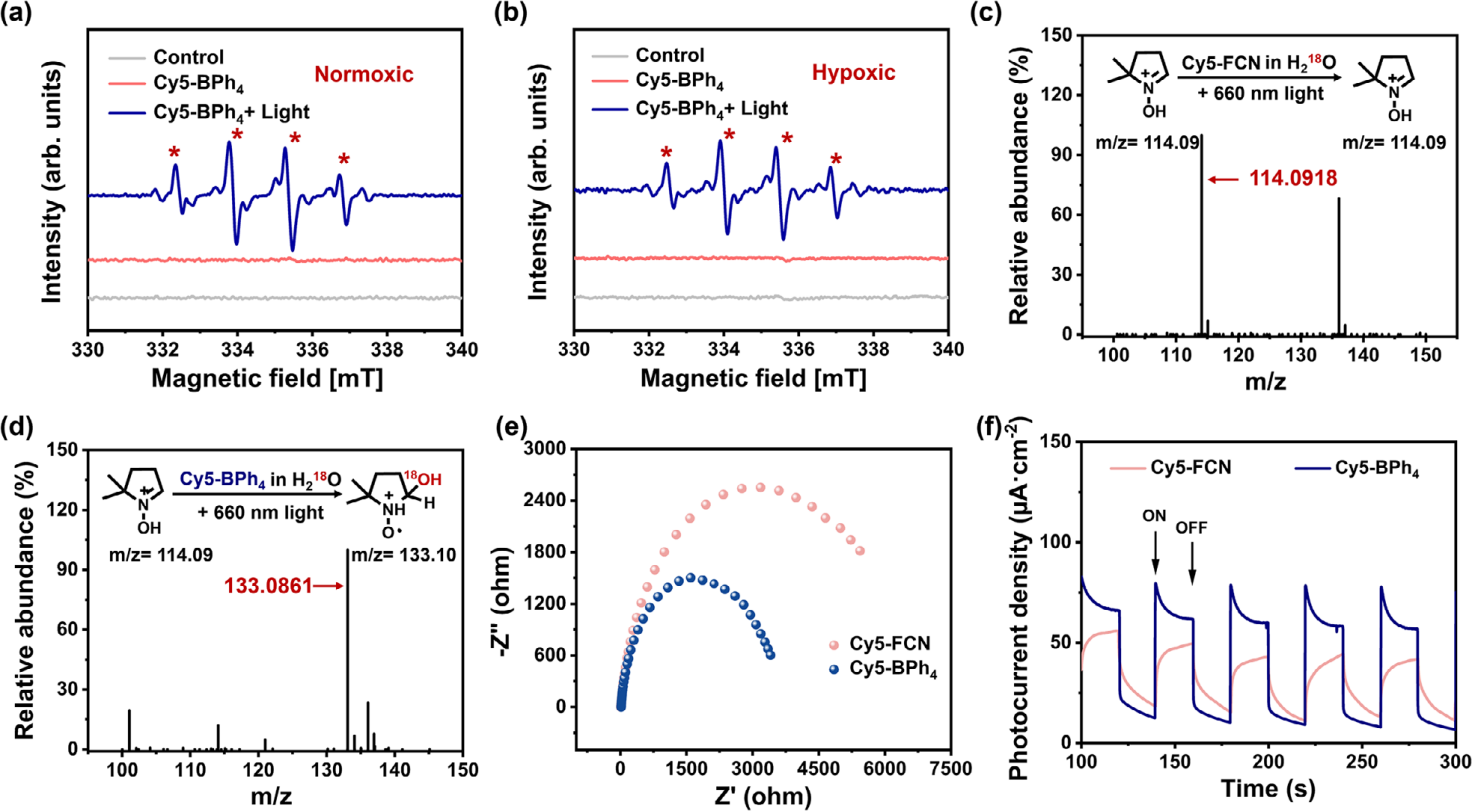
Electron transfer. The electron paramagnetic resonance (EPR) spectra of **Cy5‐BPh_4_** (0.1 mM) treated with or without light (660 nm, 100 mW cm^−2^) in redistilled water under (a) normoxic and (b) hypoxic conditions, respectively. Capturing agent: DMPO (5 mM). The mass spectra of the photoproduced •OH/DMPO adducts in (c) **Cy5‐FCN** (0.1 mM) and (d) **Cy5‐BPh_4_** (0.1 mM) utilizing H_2_^18^O as the marker of the oxygen element. (e) The electrochemical impedance spectroscopy (EIS) curves and (f) transient photocurrent responses of **Cy5‐FCN** and **Cy5‐BPh_4_**.

For investigating the excellent O_2_^•−^ and •OH generation of **PS‐BPh_4_^−^**, we took **Cy5** as an example. First, EPR assays verified the generation of O_2_^•−^ and •OH of PSs in redistilled water (Figure 3a; Figure S36), which was in line with the results in Figure 1. As is known, PSs with O_2_^•−^ and •OH generation exhibited minimal O_2_ dependency owing to the Haber‐Weiss and H_2_O oxidation process, respectively, which would confer certain advantages in hypoxic malignant solid tumor therapy [40]. Thereby, the •OH and O_2_^•−^ yields of **Cy5‐BPh_4_** in hypoxic conditions were assessed as illustrated in Figure 3b and Figure S37. Remarkably, similar resonance signal intensity of DMPO/O_2_^•−^ adducts and DMPO/•OH was observed after 660 nm laser irradiation, showcasing the efficient photoinduced O_2_^•−^ and •OH generation in **Cy5‐BPh_4_** under hypoxic conditions. To further investigate the detailed origin of the •OH generated in **Cy5‐BPh_4_**, mass spectrometry was conducted by utilizing H_2_^18^O as the solvent to mark the oxygen element. As shown in Figure 3c,d, after 660 nm light irradiation, a notable signal peak at m/z = 133.0861 (the characteristic peak of DMPO‐^18^OH) was detected in the presence of **Cy5‐BPh_4,_** while **Cy5‐FCN** only displayed a signal peak of DMPO at m/z = 114.0918 (the characteristic peak of DMPO without combining ^18^OH), proclaiming that the assembled **Cy5‐BPh_4_** could efficiently oxidize H_2_O to produce •OH under irradiation.

**FIGURE 4:**
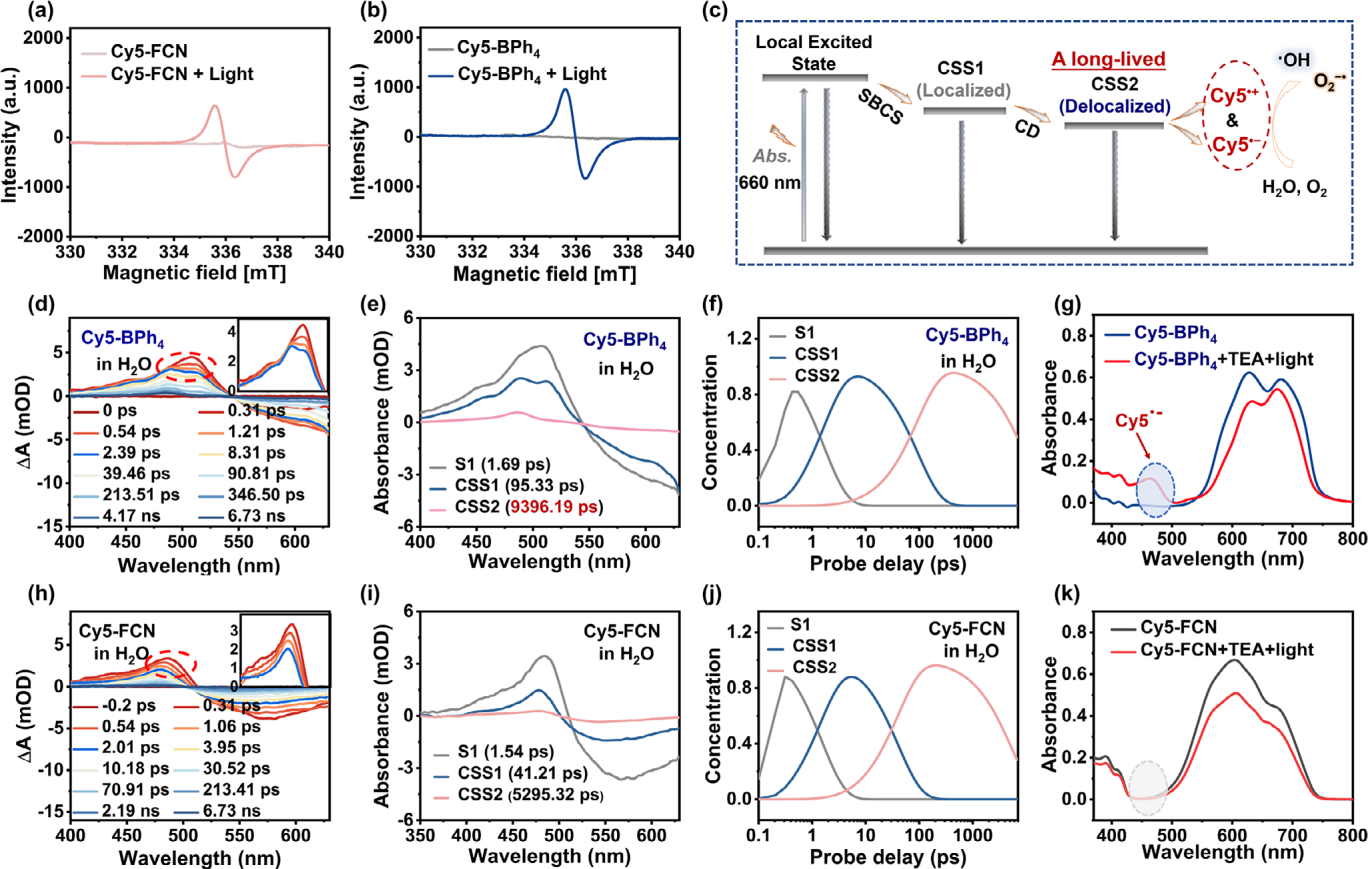
Long‐lived **CSs** of **Cy5‐BPh_4_**. (a) Electron paramagnetic resonance (EPR) spectra of electron generation for (a) **Cy5‐FCN** and (b) **Cy5‐BPh_4_** with or without light irradiation. Solid powder. (c) The mechanistic diagram of radical production by assembled cyanine PSs via a charge separation pathway. SBCS: symmetry‐breaking charge separation; CSS1: localized CSs; CD: charge delocalization; CSS2: delocalized CSs. Transient absorption spectrum (TAS) of (d) **Cy5‐BPh_4_** (10 µM) and (h) **Cy5‐FCN** (10 µM) excited at 650 nm in redistilled water. Corresponding evolution‐associated spectra (EAS) for (e) **Cy5‐BPh_4_** and (i) **Cy5‐FCN** by global fitting analysis. Corresponding concentration evolution of transient species for (f) **Cy5‐BPh_4_** and (j) **Cy5‐FCN**. The UV–vis absorption spectra for detecting the photo‐induced Cy5^•−^ production for assembled (g) **Cy5‐BPh_4_** (10 µM) and (k) **Cy5‐FCN** (10 µM) utilizing triethylamine (TEA) as an electronic capture agent upon 660 nm laser illumination.

Afterward, more in‐depth experiments were carried out to analyze the remarkable radical generation discrepancy in the two assembled PSs. Initially, as shown in Figure 3e, electrochemical impedance spectroscopy (EIS) showed that the electron transfer resistance of **Cy5‐BPh_4_** was significantly lower than that of **Cy5‐FCN**, indicating a fast surface transfer of carriers in **Cy5‐BPh_4_**. The enhanced photocurrent response of **Cy5‐BPh_4_** exhibited a more obvious electron‐ hole separating tendency in contrast to that of **Cy5‐FCN** (Figure 3f), which was beneficial for the subsequent electron transfer to generate radicals [41]. Moreover, the EPR assay revealed the photogenerated electron generation in both PSs, while **Cy5‐BPh_4_** presented a stronger signal than **Cy5‐FCN**, further confirming that the more efficient CS occurred in assembled **Cy5‐BPh_4_** (Figure 4a,b). What's more, the analogical phenomenal also observed in **MCy5‐BPh_4_**, **BCy5‐BPh_4_**, and **PCy5‐BPh_4_** (Figure S38), which proved the universality in stereo‐*π* anion **BPh_4_^−^** incorporation.

### 3.5 Excited State Dynamics for Radical Production

The results above spurred us to further explore the detailed excited‐state dynamics of distinct assembled PSs. As displayed in Figure 4d,h, at 650 nm excitation, the TAS at 0.31 ps for two PSs exhibited negative absorption peaks in redistilled water, attributed to ground‐state bleaching, which were consistent with their UV–vis absorption profiles (Figure 1b). Meanwhile, two positive absorption peaks of **Cy5‐FCN** and **Cy5‐BPh_4_** at 400–550 nm range were recorded, arising from the photon‐induced absorption (PIA) of S_1_‐S_n_ for the Cy5 skeleton (Figure S39). It is worth noting that the blue shift from 510 nm to 480 nm in TAS of **Cy5‐FCN** and **Cy5‐BPh_4_** was detected in redistilled water within 3 ps, suggesting the occurrence of intermediate species during the S1 decay process, in which the 480 nm PIA peak was assigned as the characteristic absorption of CSs for Cy5 [28]. Moreover, as illustrated in Figure 4d,h insets, two remarkable steps in the CSs feature decay process (a fast decay within 1 ns and a slow decay after 1 ns) were captured in both assembled PSs, which could be identified as the transformation from the localized CSs (CSS1) to delocalized CSs (CSS2) and the decay of CSS2. Importantly, long‐lived CSS2 with a low CR rate would facilitate the subsequent sensitization process between Cy5^•+^ / Cy5^•−^with the substrate (Figure 4c). Furthermore, the global analysis of the TAS was implemented for assembled **Cy5‐BPh_4_** and **Cy5‐FCN**, as depicted in Figure 4e,f,i,j, the **Cy5‐BPh_4_** had a longer lifetime with τ_CSS2_ of about 9396.19 ps than that of **Cy5‐FCN (**5295.32 ps). Also, triethylamine (TEA) was utilized as a cation radical sacrificial agent for detecting the Cy5^•−^ according to UV–vis spectroscopy. As shown in Figure 4g,k, the obvious 480 nm PIA feature (corresponding to the typical absorption of Cy5^•−^) was observed in the **Cy5‐BPh_4_** group, ascribed to the longer CSS2 lifetime. This fully explained the efficient radical generation capacity of assembled **Cy5‐BPh_4_** via the formation of long‐lived CSS2.

### 3.6 Mechanism Analysis for Long‐Lived CSs

**FIGURE 5:**
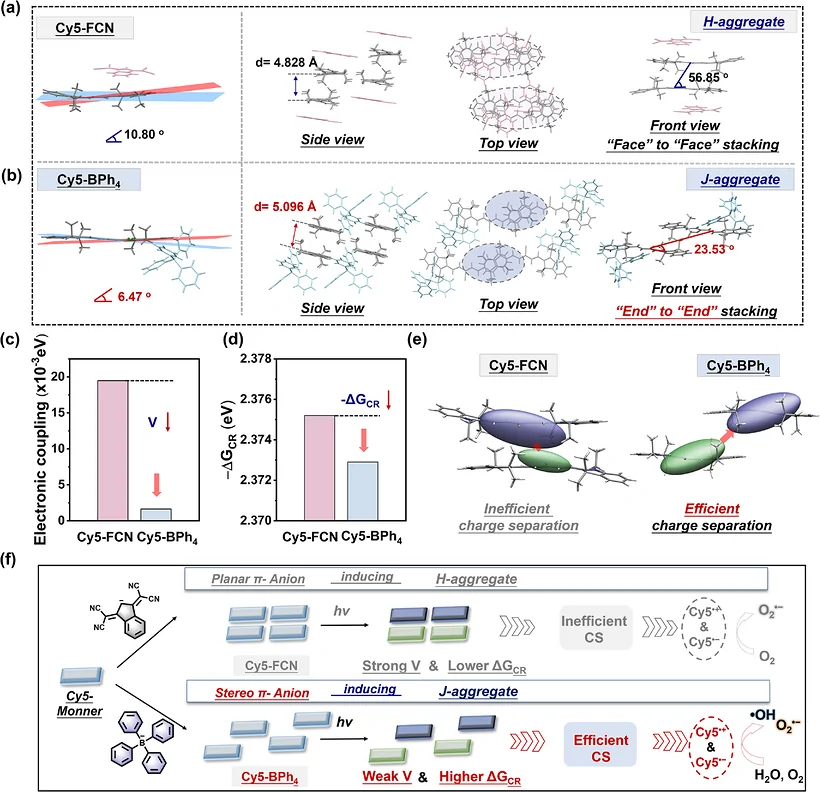
Crystallographic data and theoretical calculation of distinct assembly. Crystallographic data of (a) **Cy5‐FCN** and (b) **Cy5‐BPh_4_**. The histograms of calculated (c) electronic coupling (V) and (d) the Gibbs free energy change value of charge recombination (ΔG_CR_) for adjacent Cy5^+^ based on the M06‐2X functional with the 6–311G (d,p) basis set (GD3) and time‐dependent DFT, respectively. (e) Cele and chole of adjacent Cy5^+^ in **Cy5‐FCN** and **Cy5‐BPh_4_**. (Red arrow indicates charge transfer direction; the hole centroid points to the electron centroid.) (f) Schematic diagram of the mechanism in charge separation (CS) for radical generation that was triggered by distinct assembled behavior based on a counter‐anion strategy.

The relationship between the longer lifetime of CSs and the assembled behaviors was investigated from a molecular perspective based on the obtained single crystal. Conformational analysis in Figure 5a,b, revealed that the Cy5 cationic skeleton (Cy5^+^) in **Cy5‐BPh_4_** exhibited a more planar molecular structure with a dihedral angle of 6.47° between the indole and conjugated polyene, compared with 10.80° in **Cy5‐FCN**, which might be attributed to fewer C─H···*π* interactions formed between the stereo‐π anion **BPh_4_^−^** and Cy5^+^ in **Cy5‐BPh_4_** (Figure S40)_._ To clarify the detailed assembly behaviors affected by **FCN^−^** and **BPh_4_^−^**, we further studied the packing modes of **Cy5‐BPh_4_** and **Cy5‐FCN**. As shown in Figure 5a,b, after the incorporation of stereo‐π anion **BPh_4_^−^**, the intermolecular distance in adjacent Cy5^+^ increased from ∼ 4.828 Å (for **Cy5‐FCN**) to ∼5.096 Å (**Cy5‐BPh_4_**). Meanwhile, unlike the “Face to Face” stacking of **Cy5‐FCN** with a displacement angle (*θ*) between the adjacent Cy5^+^ of 56.85°, *J*‐aggregated **Cy5‐BPh_4_** displayed a typical “End to End” stacking with a *θ* of 23.53°. These differential conformations might be due to the significant steric hindrance effect and lateral distribution feature of **BPh_4_^−^**. The small *θ* of adjacent Cy5^+^ in **Cy5‐BPh_4_** might lead to a relatively weak V that played a crucial role in prolonging the lifetime of CSs due to the retard CR, thereby accelerating the photosensitization process in the PS systems [42].

After that, quantum chemical analysis was implemented to further disclose the effect of V on the CSs in assembled PSs. In detail, based on the crystallographic data, the pivotal physical parameters in the CS process were calculated, including V as well as ΔG_CR_, using the M06‐2X functional with the 6–311 G (d, p) basis set (GD 3) and time‐dependent DFT, respectively. As shown in Figure 5c, the *J*‐aggregates of dimeric Cy5^+^ in **Cy5‐BPh_4_** depicted lower values of V (1.6326 × 10^−3^ eV), relative to those in *H*‐aggregated **Cy5‐FCN** (V = 19.0470 × 10^−3^ eV), optimizing the CS process in assembled **Cy5‐BPh_4_** upon photoexcitation. Moreover, a more positive ΔG_CR_ (−2.3729 eV) in **Cy5‐BPh_4_** group revealed the restrained CR process compared with **Cy5‐FCN** (‐2.3753 eV), leading to the prolonged CSs lifetime according to Marcus's theory (Figure 5d). Additionally, as displayed in Figure 5e and Table 1, the electron‐hole distribution of assembled PSs was smoothed to further clarify the tendency of CS. The degree of overlap between electrons and holes (Sr) as well as the overall distribution of electrons and holes (H) between the adjacent Cy5^+^ was significantly decreased (Sr: 0.1595 vs. 0.5093; H: 4.4730 Å vs. 5.0130 Å), while the distance between the electron‐hole centroids (D) in **Cy5‐BPh_4_** (11.9400 Å) was increased relative to that of **Cy5‐FCN** (4.4740 Å), further validating the more efficient CS process in **Cy5‐BPh_4_** [43]. In short, the *J*‐aggregates **Cy5‐BPh_4_** possessed a weak V, which was conducive to hindering the CR process, favoring the formation of long‐lived CSs to yield toxic radicals (Figure 5f).

**TABLE 1:** Parameters in electron–hole analysis of **Cy5‐FCN** and **Cy5‐BPh_4_**.

Compounds	Sr^a^	H/Å^b^	D/Å^c^
**Cy5‐FCN**	0.5093	5.0130	4.4740
**Cy5‐BPh_4_**	0.1595	4.4730	11.9400

^a^ A metric that describes the overlap between the electron and hole, wherein the lower value implies a more prominent charge separation.

^b^ A parameter used to assess the overall distribution range of electrons and holes, wherein a smaller value signifies a more concentrated distribution.

^c^ Electron‐hole distance, where an increase corresponds to more obvious charge separation.

### 3.7 In Vitro Cytotoxicity and Anticancer Mechanisms

**FIGURE 6:**
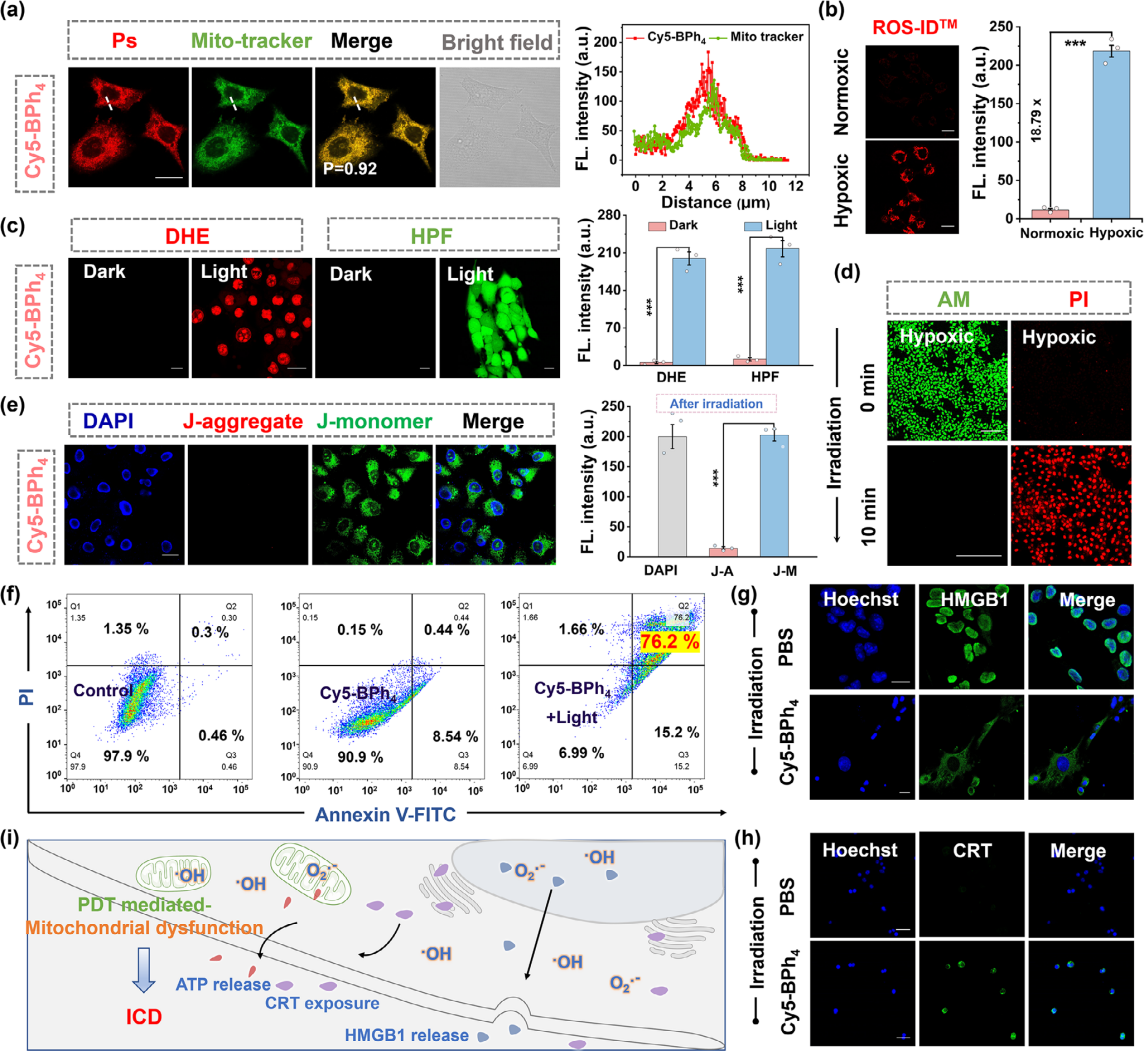
Anti‐hypoxia performance and immunogenic cell death (ICD) in cell level. (a) CLSM images of 4T1 cells pre‐treated with **Cy5‐BPh_4_** (5 µM) and Mito‐tracker Green (0.5 µM) for 10 min in the dark, along with a comparison of their fluorescence intensity in the specified region pointed by the white line. (b) CLSM images of 4T1 cells following incubation of ROS‐ID^TM^ (2 µM) (as an anaerobic indicator), and the histogram of corresponding intracellular average fluorescence intensities. (c) The detection of intracellular O_2_^•−^ and •OH generation after incubating **Cy5‐BPh_4_** for 5 min with or without light irradiation (660 nm, 100 mW cm^−2^) utilizing DHE (10 µM) and HPF (2 µM) as indicators under hypoxic conditions. Scale bar: 20 µm. (d) Live/dead co‐staining (1 µM) of 4T1 cells treated with **Cy5‐BPh_4_** with or without light irradiation under hypoxic conditions. Scale bar: 200 µm (e) CLSM images of JC‐10 (2 µM) staining in 4T1 cells following incubation of **Cy5‐BPh_4_**, DAPI (10 µM) (as a probe for nuclear), along with the quantification of the corresponding fluorescence intensity of *J‐*aggregate and *J‐*monomer. Scale bar: 20 µm. (f) Annexin V‐FITC/PI (1 µM) staining of 4T1 cells pre‐treated with **Cy5‐BPh_4_**. Immunofluorescence staining assays of (g) high mobility group box 1 (HMGB1) and (h) surface‐exposed calreticulin (CRT) of 4T1 cells with or without incubation of **Cy5‐BPh_4_** under light irradiation. Scale bar: 20 µm. (i) The graphic illustration of photodynamic therapy (PDT) ‐induced ICD process in **Cy5‐BPh_4_**. Unless otherwise specified, data throughout this study are presented as the mean ± SD. *p*‐value was calculated by one way ANOVA (^*^ *p* < 0.05, ^**^ *p* < 0.01, and ^***^ *p* < 0.001), *n* = 3.

Inspired by the superior O_2_^•−^ and •OH yield under light irradiation, **Cy5‐BPh_4_** was selected as a tumor‐targeted photodynamic agent for subsequent cellular experiments, wherein the mouse breast (4T1) and human hepatoma (Hep G2) cancer cells were used as the model cell lines. The MTT assay results (Figure S41) suggested that the cells pretreated with **Cy5‐BPh_4_** maintained high viability (over 90%) under dark conditions, demonstrating good biosafety that was suitable for the following cellular experiments. Moreover, colocalization analysis revealed efficient mitochondrial targeting of **Cy5‐BPh_4,_** with the Pearson's coefficient (Pr) of 0.92/ 0.94, when it co‐stained with Mito‐tracker Green. (Figure 6a; Figure S42). Considering that oxygen‐independent radicals had the potential to overcome the limitations of hypoxic tumors, a hypoxic environment was established to evaluate the intracellular photodynamic therapy (PDT) activity of **Cy5‐BPh**. As illustrated in Figure 6b and Figure S43, the occurrence of red fluorescence for ROS‐ID (an anaerobic indicator) confirmed the construction of hypoxic conditions. After exposure to NIR light (660 nm, 100 mW cm^−2^, 5 min), the intense cellular red fluorescence of DHE and green fluorescence of HPF were visualized both in normoxic and hypoxic condition, manifesting the superior photo‐induced yield for O_2_^•−^ and •OH by **Cy5‐BPh_4_** in vitro (Figure 6c; Figure S44), which provided a great potential for **Cy5‐BPh_4_** to effectively induce oxidative stress and ablate hypoxic cancer cells. Based on the above results, live/dead cell staining experiments (using an AM/PI kit) as well as MTT assay were conducted to offer more intuitive results of the killing effects for **Cy5‐BPh_4_** in normoxic and hypoxic environments under NIR light illumination. As shown in Figure 6d and Figures S45 and S46, the cells cultured with **Cy5‐BPh_4_** exhibited strong red fluorescence of PI regardless of whether the conditions were normoxic or hypoxic, following irradiation (660 nm, 100 mW cm^−2^, 10 min). Furthermore, MTT assays displayed in Figure S47 also confirmed the efficient therapeutic ability of **Cy5‐BPh_4_** after 10 min of irradiation. These results revealed that **Cy5‐BPh_4_** was a great phototherapeutic agent bearing efficient radical generation for eliminating cancer cells without oxygen dependence.

Given the outstanding mitochondrial targeting capability of **Cy5‐BPh_4_**, the integrity of cellular mitochondria and the potential of mitochondrial dysfunction during **Cy5‐BPh_4_**‐mediated PDT were evaluated using the JC‐10 probe. As shown in Figures S48 and S49, vivid red fluorescence of JC‐10 dyes (indicated as *J*‐aggregates) was observed in 4T1 cells pretreated with **Cy5‐BPh_4_** in the dark, indicating the integrity of the mitochondrial membrane. Conversely, under NIR laser irradiation (660 nm, 100 mW cm^−2^, 10 min), a notable shift toward green fluorescence of JC‐10 (indicated as *J*‐monomer) was observed owing to the occurrence of significant depolarization of the mitochondrial membrane potential (Figure 6e). Given that depolarized cells are prone to apoptosis, an Annexin V‐FITC/PI assay was performed to investigate cancer cell death pathways. As depicted in Figure 6f, a high apoptosis rate (76.2%) in 4T1 cells was visualized after treatment with **Cy5‐BPh_4_** and light, further confirming that the localized oxidative damage in mitochondria might initiate severe oxidative stress, ultimately causing tumor cell apoptosis.

As reported, the oxidative damage to subcellular organelles caused by the surge of photo‐generated radicals can trigger ICD with the feature of damage‐associated molecular patterns (DAMPs) release [44, 45]. Subsequently, the expression of DAMPs, including surface‐exposed calreticulin (CRT), high mobility group box 1 (HMGB1), and extracellular secretion of ATP, was investigated under 660 nm laser irradiation, followed by treatment with PBS and **Cy5‐BPh_4_**, respectively. As shown in Figure 6g, intense green fluorescence corresponding to HMGB1 was clearly observed within the nuclear region of the control groups (**PBS**, **PBS+Light**, and **Cy5‐BPh_4_**). In contrast, prominent cytoplasmic green fluorescence and negligible nuclear fluorescence were observed in the **Cy5‐BPh_4_+Light** group, indicating effective extracellular release of HMGB1 upon **Cy5‐BPh_4_+Light** treatment, which was also indexed by the enhanced extracellular HMGB1 content (Figure S50). Moreover, immunofluorescent staining assay and measurements using an ATP Assay Kit demonstrated that the **Cy5‐BPh_4_ + Light** treatment markedly increased cell‐surface CRT fluorescence as well as the extracellular secretion of ATP versus the PBS group (Figure 6h; Figure S51). These findings verified that **Cy5‐BPh_4_ + Light** treatment effectively induced the release of DAMPs.

### 3.8 Mechanisms of Antitumor Performance Against Primary and Distal Metastatic Cancers

**FIGURE 7:**
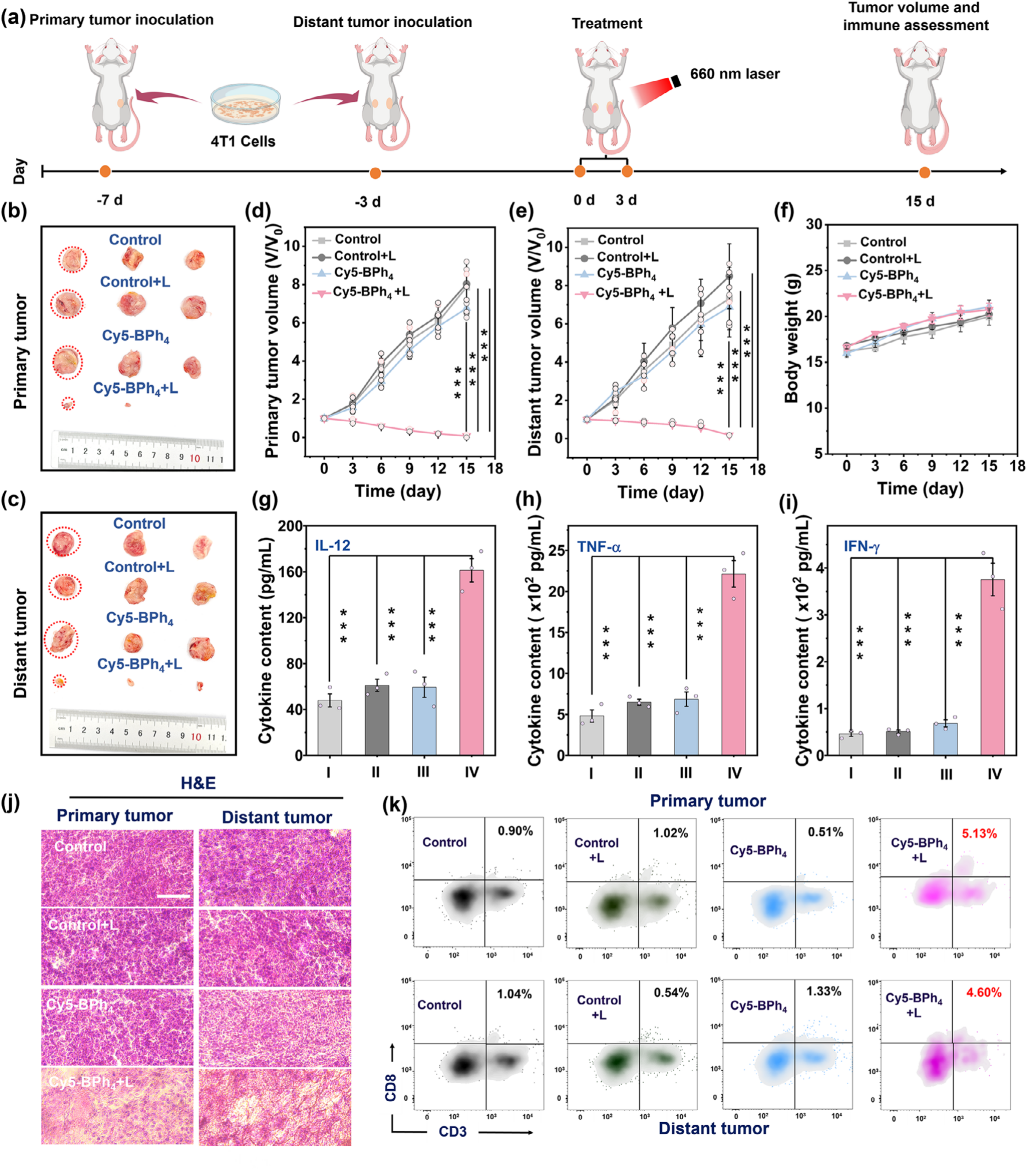
Antitumor performance and immune response. (a) Schematic diagram of experimental design for in vivo photodynamic immunotherapy mediated by **Cy5‐BPh_4_** (100 µM, 50 µL). Photographs of harvested tumors in (b) primary and (c) distant after different treatments (*n* = 3). Tumor growth curves of (d) primary and (e) distant tumors in 4T1‐tumor‐bearing mice with different treatments (*n* = 3). (f) The body weight of 4T1‐tumor‐bearing mice during the treatment. The cytokine contents, including (g) IL‐12, (h)TNF‐*α*, and (i) IFN‐ *γ* in 4T1‐tumor‐bearing mice following different treatments (*n* = 3). (j) Corresponding H&E staining photographs of primary and distant tumors in each group after 15‐day treatment (*n* = 3). (k) Flow cytometry of intra‐tumoral CD8^+^ T cell infiltration (CD3^+^ CD8^+^). Unless otherwise specified, data throughout this study are presented as the mean ± SD. *p*‐value was calculated by one way ANOVA (^*^ *p* < 0.05, ^**^ *p* < 0.01, and ^***^ *p* < 0.001), *n* = 3.

In vivo therapeutic efficacy of **Cy5‐BPh_4_** was further evaluated in female Balb/c mice implanted with subcutaneous 4T1 breast tumors, including both primary and distant tumor models, and the experimental design, as illustrated in Figure 7a. Following the primary tumors growing up to 80–100 mm^3^, the mice were randomly divided into four groups: (I) Control (PBS), (II) Control + L (PBS + Light), (III) **Cy5‐BPh_4_**, (IV) **Cy5‐BPh_4_ + L** (**Cy5‐BPh_4_ + Light**) (660 nm, 100 mW cm^−2^, 20 min). As compared to other groups, remarkable inhibition of primary tumor growth was observed after treatment with **Cy5‐BPh_4_** followed by light exposure during 15 days post‐treatment, as evidenced by the photographs of harvested tumors (Figure 7b) and the relative tumor volume measurements (Figure 7d), indicating the superior PDT effect of **Cy5‐BPh_4_**. Of note, the distant tumors were only effectively suppressed by **Cy5‐BPh_4_** under light irradiation, confirming the value of triggering immune responses (Figure 7c and e). Stable weight increases, negligible pathological discrepancies, and inflammatory damage were observed in the major organs, including the heart, liver, spleen, lung, and kidney, of mice across all groups based on H&E staining, further confirming the lack of notable systemic toxicity associated with **Cy5‐BPh_4_** administration (Figure 7f; Figure S52). Significantly, the presence of histiocytic necrosis in both primary and distant tumor tissues was clearly visualized according to H&E staining (Figure 7j), which could be attributed to the **Cy5‐BPh_4_**‐induced phototoxicity.

Moreover, more experiments were carried out to gain deeper insight into the immune response activated by the PDT effect. The dendritic cells (DCs), as the key professional antigen‐presenting cells, were initially analyzed by flow cytometry due to their critical role in regulating immune responses during the ICD process [46]. As displayed in Figure S53, in contrast to the control group (0.46%), a remarkable increase in the percentage composition of CD80^+^CD86^+^ DCs (12.4%) was observed in the **Cy5‐BPh_4_ + L** group, displaying the highest expression level of DCs. Additionally, substantial elevations in pro‐inflammatory cytokines, including IL‐12, TNF‐α, and IFN‐γ, were observed in the serum of the **Cy5‐BPh_4_ + L** group (Figure 7g–i), which corresponded with the findings on DCs maturation. These results verified that the PDT effect induced by **Cy5‐BPh_4_** effectively triggered an immune stimulation effect.

Considering that mature DCs could trigger the activation of T lymphocytes to initiate an immune response, we further evaluated the levels of cytotoxic T lymphocytes in the primary and distant tumors as well as in the spleens across different treatment groups. As descripted in Figure 7k and Figure S54, the infiltration of CD8^+^ T cells within the primary tumor of **Cy5‐BPh_4_ + L** group was 5.13%, which was notable higher than that of **Control** (0.90%), **Control + L** (1.02%), and the **Cy5‐BPh_4_** (0.51%) groups, indicating effective infiltration of CD8^+^ T cells into 4T1 tumors following treatment with **Cy5‐BPh_4_** and light exposure. Meanwhile, similar patterns of differentiation were observed in both distant tumors (4.42‐fold higher than control) and the spleen (10.24‐fold higher than control), manifesting that **Cy5‐BPh_4_**‐mediated photodynamic immunotherapy effectively promoted the differentiation of naive T cells into CD8^+^ T cells. The results demonstrated that **Cy5‐BPh_4_** could effectively reconstruct the immunosuppressive tumor microenvironment to efficiently block tumor regeneration, benefiting from boosting the cytotoxicity of radicals generated during the PDT process.

## 4 Conclusion

To sum up, the universal strategy of stereo‐*π* anion decorating was proposed to unlock the long‐lived CSs in commercial cyanine PSs for high‐yield toxic radical generation. First, a series of traditional cyanine‐based PSs (**Cy5‐X**) were developed by harnessing the stereo (**BPh_4_^−^**) and nonstereo (**OTs^−^**, **FCN^−^**) *π* anions to replace the heavy atom (I^−^). Compared with nonaggregated **Cy5‐OTs** as well as *H*‐aggregated **Cy5‐FCN**, *J*‐aggregates of **Cy5‐BPh_4_** displayed excellent O_2_^•−^ and •OH yields through an oxygen‐independent pathway. The crystal structure and theoretical calculations revealed that **Cy5‐BPh_4_** bearing a weak V held a high CS efficiency due to the small ΔG_CR_, which effectively prolonged the existence of the delocalized ion pair of PSs, thereby enhancing the validity of subsequent photoreaction. Upon irradiation, the **Cy5‐BPh_4_** aggregates not only triggered significant mitochondrial damage to cause cell apoptosis for primary tumor therapy, but also induced ICD for distant tumor treatment. It is an extremely rare report on the formation of long‐lived CSs using modulating counter‐anions, which provided a novel strategy for the fabrication of photoimmunotherapy‐PSs.

## Conflicts of Interest

The authors declare no conflicts of interest.

## Supporting information




**Supporting File**: advs77556‐sup‐0001‐SuppMat.docx.

## Data Availability

The data that support the findings of this study are available from the corresponding author upon reasonable request.
