# Supplementary material for: Unlocking Long‐Lived Charge‐Separated State in Pentamethine Cyanine Based on Anionic Hofmeister Effect for Photodynamic Immunotherapy
Source: Adv Sci (Weinh). 2026 Sep 11:e77556. Online ahead of print. doi: 10.1002/advs.77556 (PMC13564192; doi:10.1002/advs.77556)
Supplement: Supplementary file 1 [file ADVS-9999-e77556-s001.docx]

**Supporting Information**

**Unlocking Long-lived Charge Separated State in Pentamethine Cyanine Based on Anionic Hofmeister Effect for Photodynamic Immunotherapy**

Xuan Zhao^1 a^, Lei Jiang^1 a^, Yingyong Ni^*^ ^a^, Ting Wang ^a^, Junjun Wang^*^ ^c^, Shengyu Shi ^b^, Jianhua Yu ^a^, Zhou Lu ^e^, Xiaojiao Zhu^*^ ^a^, Hongping Zhou^* a, b^

*^a^* *School of Chemistry and Chemical Engineering, School of Materials Science and Engineering, Center of Free Electron Laser & High Magnetic Field, Key Laboratory of Structure and Functional Regulation of Hybrid Materials Ministry of Education, Key Laboratory of Functional Inorganic Materials Chemistry of Anhui Province, Key Laboratory of Chemistry for Inorganic/Organic Hybrid Functionalized Materials of Anhui Province, Anhui University, Hefei 230601, P. R. China.*

*^b^ College of Biological and Food Engineering, School of Chemical and Environmental Engineering, Anhui Polytechnic University, 241000 Wuhu, PR China.*

*^c^Department of Chemistry and Chemical Engineering, Anhui Engineering Laboratory for Medicinal and Food Homologous Natural Resources Exploration, Hefei Normal University, Hefei 230061, China.*

*^d^ School of Physics and Electronic Information, Anhui Normal University, 241002 Wuhu, PR China.*

*Corresponding author.

**Email:** [zhpzhp@263.net](mailto:zhpzhp@263.net) (Hongping Zhou), [18895636492@163.com](mailto:18895636492@163.com) (Yingyong Ni), [wangjunjun@hfnu.edu.cn](mailto:wangjunjun@hfnu.edu.cn) (Junjun Wang), [xiaojzhu@ahu.edu.cn](mailto:xiaojzhu@ahu.edu.cn) (Xiaojiao Zhu).

**Content**

[1. Synthetic Routes and Characterization	3](#_Toc225934831)

[1.1 Synthesis of Cy5-OTs:	3](#_Toc225934832)

[1.2 Characteristics of Cy5-FCN:	4](#_Toc225934833)

[1.3 Characteristics of Cy5-BPh4:	4](#_Toc225934834)

[1.4 Characteristic of MCy5-BPh_4_:	5](#_Toc225934835)

[1.5 Characteristic of BCy5-BPh_4_:	5](#_Toc225934836)

[1.6 Characteristic of PCy5-BPh_4_:	5](#_Toc225934837)

[2. Supporting Figures.	6](#_Toc225934838)

[3. Supporting Table	29](#_Toc225934839)

[4. Reference	29](#_Toc225934840)

# 1. Synthetic Routes and Characterization

**Scheme S1.** Synthesis routes of **Cy5-OTs**, **Cy5-FCN**, and **Cy5-BPh_4_**.

Compound **Cy5-I** was synthesized according to reported works. ^[S1]^

1.1 Synthesis of Cy5-OTs: In the dark conditions, **Cy5-I** (0.10 g, 0.16 mmol) and silver nitrate (0.047 g, 0.17 mmol) were added to a 100 mL single-neck round-bottom flask with methanol (20 mL) and refluxed for 5 hours at 65 °C. After completion of the reaction, the mixture was cooled to room temperature and filtered; the solvent was then removed under reduced pressure. The required **Cy5-OTs** was obtained as a dark solid in 82.7 % yield. ^1^H NMR (400 MHz, DMSO-*d*_6_) *δ*(ppm): 8.47 (d, *J* = 12.1 Hz, 2 H), 7.65 (d, *J* = 7.4 Hz, 2 H), 7.48 (d, *J* = 8.0 Hz, 2 H), 7.44-7.39 (m, 4 H), 7.29 (t, *J* = 7.4 Hz, 2 H), 7.06 (d, *J* = 8.0 Hz, 2 H), 6.29 (d, *J* = 13.4 Hz, 2 H), 4.17 (q, *J* = 7.2 Hz, 2 H), 2.24 (s, 3 H), 1.68 (s, 13 H), 1.29 (t, *J* = 7.1 Hz, 2 H). ^13^C NMR (101 MHz, DMSO-*d*_6_) *δ* (ppm):173.8, 149.5, 145.9, 141.5, 137.5, 128.6, 128.0, 125.5, 122.6, 115.5, 101.6, 49.5, 26.57, 21.5, 20.8, 11.9, 4.0. FTMS (ESI): calcd for: C_29_H_34_BrN_2_^+^ [M]^+^, 490.51; found, 489.1879. ITMS (ESI): calcd for: C_15_H_5_N_4_^-^ [M]^-^, 171.02; found, 171.13.

1.2 Characteristic of Cy5-FCN: ^1^H NMR (400 MHz, DMSO-*d*_6_) *δ*(ppm): 8.51 (d, *J* = 13.4 Hz, 2 H), 7.91 (dd, *J_1_* = 5.9 Hz, *J_2_* = 3.1 Hz, 2 H), 7.69 (d, *J* = 7.4 Hz, 2 H), 7.52 (d, *J* = 8.0 Hz, 2 H), 7.43 (dd, *J_1_* = 5.6 Hz, *J_2_* = 3.1 Hz, 2 H), 7.33 (t, *J* = 7.4 Hz, 2 H), 6.33 (d, *J* = 13.4 Hz, 2 H), 5.71 (s, 1 H), 4.22 (q, *J* = 7.1 Hz, 4 H), 1.72 (s, 12H), 1.34 (t, *J* = 7.1 Hz, 6 H). ^13^C NMR (101 MHz, DMSO-*d*_6_) *δ*(ppm):174.4, 158.7, 150.1, 142.1, 141.9, 130.6, 129.1, 126.1, 123.2, 122.1, 118.5, 118.4, 116.1, 112.1, 103.3, 102.2, 50.9, 50.0, 27.6, 12.5. FTMS (ESI): calcd for: C_29_H_34_BrN_2_^+^ [M]^+^, 490.51; found, 491.1895. ITMS (ESI): calcd for: C_15_H_5_N_4_^-^ [M]^-^, 241.05; found, 241.13.

1.3 Characteristic of Cy5-BPh_4_: ^1^H NMR (400 MHz, DMSO-*d*_6_) *δ*(ppm): 8.47 (d, *J* = 13.5 Hz, 2 H), 7.65 (d, *J* = 7.4 Hz, 2 H), 7.47 (d, *J* = 7.9 Hz, 2 H), 7.41 (t, *J* = 7.4 Hz, 2 H), 7.28 (t, *J* = 7.4 Hz, 2 H), 7.14 (t, *J* = 7.2 Hz, 8 H), 6.88 (t, *J* = 7.4 Hz, 8 H), 6.74 (t, *J* = 7.2 Hz, 4 H), 6.29 (d, *J* = 13.4 Hz, 2 H), 4.16 (q, *J* = 7.3 Hz, 4 H), 1.68 (s, 12H), 1.29 (t, *J* = 7.1 Hz, 6 H). ^13^C NMR (101 MHz, DMSO-*d*_6_) *δ*(ppm):174.4, 164.4, 164.1, 163.8, 163.4, 150.1, 142.1, 141.9, 136.1, 129.2, 126.2, 125.9, 125.8, 123.2, 22.1, 116.1, 112.1,102.2, 50.1, 27.2, 12.5. FTMS (ESI): calcd for: C_29_H_34_BrN_2_^+^ [M]^+^, 490.51; found, 491.1895. ITMS (ESI): calcd for: C_24_H_20_B^-^ [M]^-^, 319.17; found, 319.23.

 **Scheme S2.** Synthesis routes of **MCy5-BPh_4_**, **BCy5-BPh_4_**, and **PCy5-BPh_4_**.

The synthesis methods of compounds **MCy5-BPh_4_**, **BCy5-BPh_4_**, and **PCy5-BPh_4_** were similar to that of **Cy5-BPh_4_**.

1.4 Characteristic of MCy5-BPh_4_: ^1^H NMR (400 MHz, DMSO-*d*_6_) *δ*(ppm): 8.45 (d, *J* = 13.4 Hz, 2H), 7.63 (d, *J* = 7.5 Hz, 2H), 7.46 (d, *J* = 8.0 Hz, 2H), 7.40 (t, *J* = 7.5 Hz, 2H), 7.27 (t, *J* = 7.3 Hz, 2H), 7.13 (t, *J* = 6.6 Hz, 8H), 6.88 (t, *J* = 7.4 Hz, 8H), 6.74 (t, *J* = 7.2 Hz, 4H), 6.26 (d, *J* = 13.4 Hz, 2H), 3.63(s, 6H), 1.68(s, 12H). ^13^C NMR (101 MHz, DMSO-*d*_6_) *δ*(ppm): 175.2, 149.7, 143.1, 141.8, 136.1, 129.0, 126.0, 125.8, 123.0, 122.1, 116.0, 112.3, 102.7, 49.9, 32.0, 27.1. FTMS (ESI): calcd for: C_27_H_30_BrN_2_^+^ [M]^+^, 462.50; found, 463.1568. ITMS (ESI): calcd for: C_24_H_20_B^-^ [M]^-^, 319.17; found, 319.24.

1.5 Characteristic of BCy5-BPh_4_: ^1^H NMR (400 MHz, DMSO-*d*_6_) *δ*(ppm): 8.51 (d, *J* = 13.4 Hz, 2H), 7.69 (d, *J* = 7.4 Hz, 2H), 7.52 (d, *J* = 8.0 Hz, 2H), 7.45 (t, *J* = 7.5 Hz, 2H), 7.32 (t, *J* = 7.5 Hz, 2H), 7.20-7.16 (m, 8H), 6.92 (t, *J* = 7.4 Hz, 8H), 6.78 (t, *J* = 8.5 Hz, 4H), 6.36 (d, *J* = 13.5 Hz, 2H), 4.17 (t, *J* = 7.2 Hz, 4H), 1.79-1.72 (m, 16H), 1.45-1.38 (m, 4H), 0.96 (t, *J* = 7.4 Hz, 6H). ^13^C NMR (101 MHz, DMSO-*d*_6_) *δ*(ppm): 174.8, 164.6, 164.1, 163.6, 163.1, 149.8, 142.3, 141.9, 135.5, 126.1, 125.7, 123.1, 122.0, 116.15, 112.3, 102.6, 50.0, 44.2, 29.4, 27.1, 20.1, 14.3. FTMS (ESI): calcd for: C_33_H_42_BrN_2_^+^ [M]^+^, 545.30; found, 545.25. ITMS (ESI): calcd for: C_24_H_20_B^-^ [M]^-^, 319.17; found, 319.22.

1.6 Characteristic of PCy5-BPh_4_: ^1^H NMR (400 MHz, DMSO-*d*_6_) *δ*(ppm): 8.67 (d, *J* = 13.4 Hz, 2H), 8.48 (d, *J* = 8.6 Hz, 2H), 8.18 (q, *J* = 8.9 Hz, 4H), 7.84 (d, *J* = 9.0 Hz, 2H), 7.72 (t, *J* = 8.4 Hz, 2H), 7.56 (t, *J* = 7.1 Hz, 2H), 7.20-7.71 (m, 8H), 6.92 (t, *J* = 7.4 Hz, 8H), 6.78 (t, *J* = 7.1 Hz, 4H), 6.40 (d, *J* = 13.5 Hz, 2H), 4.34 (q, *J* = 7.3 Hz, 4H), 2.00 (s, 12H), 1.40 (t, *J* = 5.9 Hz, 6H). ^13^C NMR (101 MHz, DMSO-*d*_6_) *δ*(ppm): 175.3, 164.6, 164.1, 163.6, 163.1, 149.0, 139.6, 135.6, 134.4, 132.2, 131.0, 130.5, 128.4, 128.0, 125.7, 122.7, 122.0, 116.1, 112.2, 101.9, 51.8, 26.8, 12.7. FTMS (ESI): calcd for: C_37_H_38_BrN_2_^+^ [M]^+^, 589.20; found, 589.2220. ITMS (ESI): calcd for: C_24_H_20_B^-^ [M]^-^, 319.17; found, 319.29.

# 2. Supporting Figures.

**Figure S1.** ^1^H NMR spectrum of **Cy5-I** in DMSO-*d_6_*.

**Figure S2.** ^13^C NMR spectrum of **Cy5-I** in DMSO-*d_6_*.

**Figure S3.** ESI- FTMS spectrum of **Cy5-I** in MeOH.

**Figure S4.** ESI-ITMS spectrum of **Cy5-I** in MeOH.

**Figure S5.** ^1^H NMR spectrum of **Cy5-OTs** in DMSO-*d_6_*.

**Figure S6.** ^13^C NMR spectrum of **Cy5-OTs** in DMSO-*d_6_*.

**Figure S7.** ESI- FTMS spectrum of **Cy5-OTs** in MeOH.

**Figure S8.** ESI- FTMS spectrum of **Cy5-OTs** in MeOH.

**Figure S9.** ^1^H NMR spectrum of **Cy5-FCN** in DMSO-*d_6_*.

**Figure S10.** ^13^C NMR spectrum of **Cy5-FCN** in DMSO-*d_6_*.

**Figure S11.** ESI- FTMS spectrum of **Cy5-FCN** in MeOH.

**Figure S12.** ESI- ITMS spectrum of **Cy5-FCN** in MeOH.

**Figure S13.** ^1^H NMR spectrum of **Cy5-BPh_4_** in DMSO-*d_6_*.

**Figure S14.** ^13^C NMR spectrum of **Cy5-BPh_4_** in DMSO-*d_6_*.

**Figure S15.** ESI- FTMS spectrum of **Cy5-BPh_4_** in ACN.

**Figure S16.** ESI- ITMS spectrum of **Cy5-BPh_4_** in ACN.

**Figure S17.** ^1^H NMR spectrum of **MCy5-BPh_4_** in DMSO-*d_6_*.

**Figure S18.** ^13^C NMR spectrum of **MCy5-BPh_4_** in DMSO-*d_6_*.

**Figure S19.** ESI- FTMS spectrum of **MCy5-BPh_4_** in ACN.

**Figure S20.** ESI- ITMS spectrum of **MCy5-BPh_4_** in ACN.

**Figure S21.** ^1^H NMR spectrum of **BCy5-BPh_4_** in DMSO-*d_6_*.

**Figure S22.** ^13^C NMR spectrum of **BCy5-BPh_4_** in DMSO-*d_6_*.

**Figure S23.** ESI- FTMS spectrum of **BCy5-BPh_4_** in ACN.

**Figure S24.** ESI- ITMS spectrum of **BCy5-BPh_4_** in ACN.

**Figure S25.** ^1^H NMR spectrum of **PCy5-BPh_4_** in DMSO-*d_6_*.

**Figure S26.** ^13^C NMR spectrum of **PCy5-BPh_4_** in DMSO-*d_6_*.

**Figure S27.** ESI- FTMS spectrum of **PCy5-BPh_4_** in ACN.

**Figure S28.** ESI- ITMS spectrum of **PCy5-BPh_4_** in ACN.

**Figure S29.** The fluorescence spectra of **Cy5-I**, **Cy5-OTs**, **Cy5-FCN** and **Cy5-BPh_4_** in DMSO and redistilled water.

**Figure S30.** DLS analysis of (a) **Cy5-I** and (c) **Cy5-OTs** in aqueous solution. The representative TEM images of (b) **Cy5-I** and (d) **Cy5-OTs**. Scale bar: 200 nm.

**Figure S31.** The fluorescence spectra of DCFH-DA after incubating with (a) redistilled water (Control), (b) **Cy5-I,** (c) **Cy5-OTs**, (d) **Cy5-FCN**, and (e) **Cy5-BPh_4_** under 660 nm laser irradiation (100 mW cm^−2^) within 30 s for detecting total ROS generation, respectively.

**Figure S32.** The absorption spectra of ABDA after incubating with (a) redistilled water (Control), (b) **Cy5-I,** (c) **Cy5-OTs**, (d) **Cy5-FCN**, and (e) **Cy5-BPh_4_** under 660 nm laser irradiation (100 mW cm^−2^) within 100 s for detecting ^1^O_2_ generation, respectively.

**Figure S33.** The fluorescence spectra of DHR123 after incubating with (a) redistilled water (Control), (b) **Cy5-I,** (c) **Cy5-OTs**, (d) **Cy5-FCN**, and (e) **Cy5-BPh_4_** under 660 nm laser irradiation (100 mW cm^−2^) within 30 s for detecting O_2_^•−^ generation, respectively.

**Figure S34.** The fluorescence spectra of HPF after incubating with (a) redistilled water (Control), (b) **Cy5-I,** (c) **Cy5-OTs**, (d) **Cy5-FCN**, and (e) **Cy5-BPh_4_** under 660 nm laser irradiation (100 mW cm^−2^) within 30 s for detecting •OH generation, respectively.

**Figure S35.** The fluorescence spectra of DHR123 after incubating with (a) **MCy5-I**, (b) **BCy5-I**, and (c) **PCy5-I** under 660 nm laser irradiation (100 mW cm^−2^) within 60 s for detecting O_2_^•−^ generation, respectively. The fluorescence spectra of HPF after incubating with (d) **MCy5-I**, (e) **BCy5-I**, and (f) **PCy5-I** under 660 nm laser irradiation (100 mW cm^−2^) within 60 s for detecting •OH generation, respectively.

**Figure S36.** The EPR spectra of **Cy5-I**, **Cy5-OTs**, **Cy5-FCN**, and **Cy5-BPh_4_** treated with light (660 nm, 100 mW cm^−2^, 1 min) in (a) MeOH and (b) redistilled water for detecting O_2_^•−^ and •OH, capturing agent: DMPO.

**Figure S37.** The EPR spectra of **Cy5-BPh_4_** treated with light or without light (660 nm, 100 mW cm^−2^, 1 min) under hypoxia in MeOH, capturing agent: DMPO.

**Figure S38.** EPR spectra of electron generation for (a) **MCy5- BPh_4_**, (b) **BCy5- BPh_4_**, and (c) **PCy5- BPh_4_** with or without light irradiation.

**Figure S39.** Fs-TA spectra of **Cy5-I** excited at 650 nm in redistilled water.

**Figure S40.** Crystallographic data of **Cy5-FCN** and **Cy5-BPh_4_**.

**Figure S41.** Assessment of cell viability with different concentrations of **Cy5-BPh_4_** treatment without light irradiation.

**Figure S42.** CLSM images of Hep G2 cells pretreated with **Cy5-BPh_4_** and Mito-tracker Green for 10 min in the dark, along with a comparison of their fluorescence intensity in the specified region pointed by the white line. Scale bar: 20 μm.

**Figure S43.** CLSM images of Hep G2 cells following the incubation of ROS-ID^TM^ under normoxic and hypoxic conditions, respectively. Scale bar: 20 μm.

**Figure S44.** The detection of intracellular O_2_^•−^ and •OH generation in (a) 4T1 cells under normoxic conditions after incubating **Cy5-BPh_4_** and PBS for 5 min, and (b) Hep G2 cells under normoxic and hypoxic conditions after incubating **Cy5-BPh_4_** for 5 min with or without light irradiation (660 nm, 100 mW cm^−2^) utilizing DHE and HPF as indicators. Scale bar: 20 μm.

**Figure S45.** Live/dead co-staining of 4T1 cells treated with PBS in dark and light under normoxic conditions (660 nm, 100 mW cm^−2^). Scale bar: 200 μm.

**Figure S46.** Live/dead co-staining of 4T1 cells treated with **Cy5-BPh_4_** in dark and light under normoxic condition (660 nm, 100 mW cm^−2^). Scale bar: 200 μm.

**Figure S47.** Assessment of cell viability in (a) 4T1 and (b) Hep G2 cells with different concentrations of **Cy5-BPh_4_** treatment upon light irradiation under normoxic and hypoxic condition.

**Figure S48.** CLSM images of JC-10 staining in 4T1 cells following incubation with PBS and DAPI (as a probe for nuclear) with light irradiation. Scale bar: 20 μm.

**Figure S49.** CLSM images of JC-10 staining in 4T1 cells following the incubation of **Cy5-BPh_4_** and DAPI (as a probe for nuclear) without light irradiation. Scale bar: 20 μm.

**Figure S50.** Extracellular concentrations of HMGB1 after treating the 4T1 cells with I) **PBS**, II) **PBS + Light**, III) **Cy5-BPh_4_**, and IV) **Cy5-BPh_4_ + Light** (600 nm laser, 100 mW cm^-2^, 10 min).

**Figure S51.** The luminescence of extracellular ATP after treating the 4T1 cells with I) **PBS**, II) **PBS + Light**, III) **Cy5-BPh_4_**, and IV) **Cy5-BPh_4_ + Light**. (600 nm laser, 100 mW cm^-2^, 10 min).

**Figure S52.** Images of major organs (heart, liver, spleen, lungs and kidneys) of mice stained with H&E Kits in each group.

**Figure S53.** Flow cytometry of the intra-lymph node CD80^+^CD86^+^ DCs with different treatments.

**Figure S54.** Flow cytometry of intra-spleen CD8^+^ T cell infiltration (CD3^+^ CD8^+^) with different treatments.

# 3. Supporting Table

**Table S1** Crystal data and structure refinement for the **Cy5-FCN** and **Cy5-BPh_4_** (CCDC: 2478812, CCDC: 2478811)

| **Compound** | **Cy5-FCN** | **Cy5-BPh_4_** |
| --- | --- | --- |
| **Formula weight** | 731.72 | 809.70 |
| **Crystal system** | monoclinic | monoclinic |
| **Space group** | P 2_1_/c | P 2_1_/c |
| **a/Å** | 12.196(4) | 20.688(2) |
| **b/Å** | 32.466(11) | 10.9003(11) |
| **c/Å** | 9.724(3) | 19.787(2) |
| ***α*/°** | 90 | 90 |
| ***β*/°** | 97.921(5) | 90.3630(10) |
| ***γ*/°** | 90 | 90 |
| **Volume/Å^3^** | 3813(2) | 4462.0(8) |
| ***Z*** | 4 | 4 |
| ***ρ*_calc_g/cm^3^** | 1.275 | 1.205 |
| ***μ*/mm^‑1^** | 1.117 | 0.958 |
| ***F*(000)** | 1520.0 | 1704.0 |
| **Radiation** | Mo K*α* (*λ* = 0.71073) | Mo K*α* (*λ* = 0.71073) |
| **Index ranges** | -14 ≤ *h* ≤ 13*,* -38 ≤ *k* ≤ 38,  -11 ≤ *l* ≤ 11 | -26 ≤ *h* ≤ 26, -13 ≤ *k* ≤ 13, -24 ≤ *l* ≤ 25 |
| **Final R indexes [I>=2*σ* (I)]** | R_1_ = 0.0660, wR_2_ = 0.1356 | R_1_ = 0.0382, wR_2_ = 0.0925 |

# 4. Reference

[S1] H. Huang, S. Long, M. Li, F. Gao, J. Du, J. Fan, X. Peng, Dyes Pigment. 2018, *149*, 633.
